# Impact of Surface Ligand Identity and Density on the Thermodynamics of H Atom Uptake at Polyoxovanadate-Alkoxide Surfaces

**DOI:** 10.1021/acs.inorgchem.3c04435

**Published:** 2024-04-09

**Authors:** Kathryn R. Proe, Andreas Towarnicky, Alex Fertig, Zhou Lu, Giannis Mpourmpakis, Ellen M. Matson

**Affiliations:** † Department of Chemistry, University of Rochester, Rochester, New York 14627, United States; ‡ Department of Chemical Engineering, University of Pittsburgh, Pittsburgh, Pennsylvania 15261, United States

## Abstract

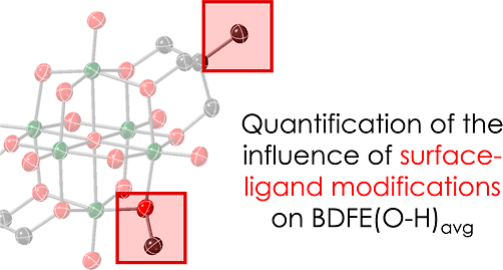

An understanding of how molecular structure influences the thermodynamics of H atom transfer is critical to designing efficient catalysts for reductive chemistries. Herein, we report experimental and theoretical investigations summarizing structure–function relationships of polyoxovanadate-alkoxides that influence bond dissociation free energies of hydroxide ligands located at the surface of the cluster. We evaluate the thermochemical descriptors of O–H bond strength for a series of clusters, namely [V_6_O_13−*x*_(OH)_*x*_(TRIOL^R^)_2_]^−2^ (*x* = 2, 4, 6; R = NO_2_, Me) and [V_6_O_11–*x*_(OMe)_2_(OH)_*x*_(TRIOL^NO2^)_2_]^−2^, via computational analysis and open circuit potential measurements. Our findings reveal that modifications to the TRIOL ligand (e.g., changing from the previously reported electron withdrawing nitro-backed ligand to the electron-donating methyl variant) have limited influence on the strength of surface O–H bonds as a result of near complete thermodynamic compensation in these systems (i.e., correlated changes in redox potential and cluster basicity). In contrast, changes in surface density of alkoxide ligands via direct alkoxylation of the polyoxovanadate-alkoxide surface result in measurable increases in bond dissociation free energies of surface O–H bonds for the mixed-valent derivatives. Our findings indicate that the extent of (de)localization of electron density across the cluster core has an impact on the bond dissociation free energies of surface O–H bonds across all oxidation states of the assembly.

## Abstract 2

Consequences of modifications made to the ligand identity and density of polyoxovanadate-alkoxide clusters on the thermodynamics of H atom uptake are investigated. Modifications to the TRIOL ligand have limited influence on the strength of surface O–H bonds as a result of thermodynamic compensation in these systems. In contrast, a change in surface density of ligands at the polyoxovanadate-alkoxide surface results in measurable increases in bond dissociation free energies of O–H bonds.

## Introduction

Charge transfer reactions at the surface of colloidal transition metal oxides are central to their function in emergent energy conversion and storage technologies (e.g., (photo)electrocatalysis, pseudocapacitors, batteries, etc.).^1−7^ While historically, redox reactions at the surface of colloidal nanomaterials have been considered purely as electron transfer processes, there is increasing evidence that the stoichiometry, thermodynamics, and kinetics of interfacial charge transfer are dictated by the identity of the charge-compensating cations.^7^ In the case of protons, the concerted transfer of proton and electron equivalents to the surfaces of redox–active transition metal oxides results in the formation of reactive H atom equivalents poised for reduction chemistries. As such, there is interest in elucidating the thermodynamics of H atom transfer at the surface of redox active transition metal oxides, parametrized as the bond dissociation free energies of the resultant hydroxyl substituents (BDFE(O–H)).^7−11^

Given the significance of bond dissociation free energies in dictating the reactivity of H atom equivalents located at the surface of transition metal oxides, there is interest in controlling this thermodynamic parameter through modifications to the material. Approaches to engineering the thermochemistry of H atom uptake and transfer include heterometal doping, the formation of O atom vacancies (i.e., anionic doping),^9,12^ and the generation of bulk defects via the deposition of heteromaterials (e.g., nanoparticles, thin films).^13^ Comparatively, less is known about how the density and identity of surface ligands influence the thermochemistry of H atom transfer reactions. This gap in knowledge is striking, as organic ligands at the surface of colloidal nanoparticles have been shown to play a significant role in the stability and reactivity of these materials.^6^

In an effort to understand the site-specific thermochemistry at the dynamic surface of metal oxide materials, our research team has turned to polyoxometalates as models for H atom uptake and transfer reactions. As summarized in our recent Account,^14^ atomically precise polyoxovanadate-alkoxide (POV-alkoxide) clusters act as models for redox reactions at solid state transition metal oxide materials without complications from cation intercalation and heterogeneity in material composition. This work has provided experimental evidence that hydrogen atoms bound at both bridging and terminal metal oxide sites behave as H atom-relay small molecule substrates.

**Figure 1:**
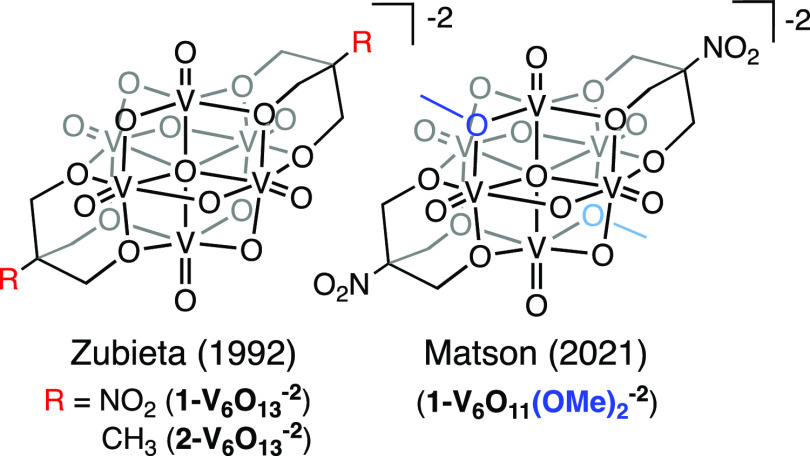
Overview of clusters studied in this work.

To further investigate the role that ligands play in controlling the thermodynamics of H atom adsorption at the surface of vanadium oxide clusters, we turned to a seminal report by Zubieta,^15^ in which a family of Lindqvist-type POV-alkoxide clusters is introduced (Figure 1). These clusters feature alternating bridging and terminal oxide sites available for protonation and are able to access a range of oxidation states. The original report demonstrated that the redox properties of the metal center of this hexavanadate cluster with the general formula, [V_6_O_13_(TRIOL^R^)_2_]^−2^ (TRIOL = tris(hydroxymethyl)methane; R = Me, Et, CH_2_OH, NO_2_, NMe_2_), could be tuned as a function of the electron-withdrawing or electron-donating properties of the functional group bound to the TRIOL ligand scaffold. Further work from our group has demonstrated the ability to substantially alter the electrochemical profile of these clusters via the sequential functionalization of bridging oxides with methyl substituents.^16^ Given the significant changes in reduction potential of these clusters observed upon surface functionalization, we hypothesized that the thermochemistry of H atom uptake and transfer at the surfaces of clusters would likewise be affected.

Herein, we demonstrate deviations in BDFE(O–H)_avg_ of surface hydroxide ligands that occur following modification of both oxidation state distribution and direct surface functionalization of the POV-alkoxide cluster. Furthermore, we provide computational analysis to support the experimentally observed phenomena. Overall, this work establishes structure–function relationships with implications for the rational design of future catalysts, offering concrete handles to optimize the proton-coupled electron transfer (PCET) chemistry at these metal oxide interfaces.

## Results and Discussion

### Effect of Oxidation State on the BDFE(O–H)_avg_ of Reduced POV-Alkoxide Clusters

**Scheme 1:**
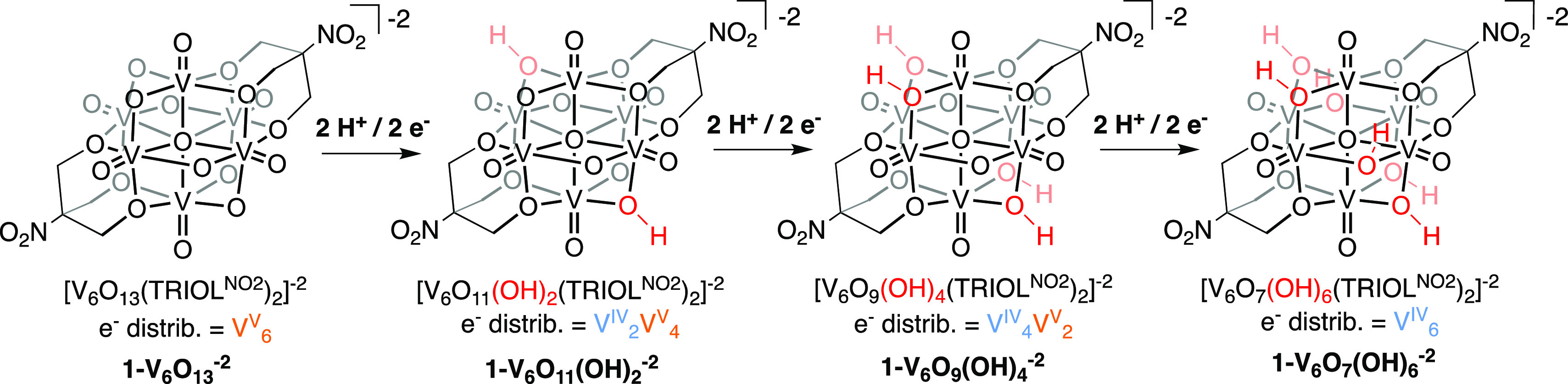
H-Atom Uptake at **1-V**_**6**_**O**_**13**_^**–2**^; Access to Reduced Forms of the Lindqvist-Type POV-Alkoxide Cluster via Proton Coupled Electron Transfer The O–H positions shown are per XRD results and may be taken as illustrative, not exclusive of other possible reaction product O–H positions.

Previously, we have described H atom uptake and transfer from the surface of a series of reduced POV-alkoxide clusters with the general formula [V_6_O_13–*x*_(OH)_*x*_(TRIOL^NO2^)_2_]^−2^, where *x* = 0, 2, 4, and 6 (**1-V**_**6**_**O**_**13**_^**–2**^, **1-V**_**6**_**O**_**11**_**(OH)**_**2**_^**–2**^, **1-V**_**6**_**O**_**9**_**(OH)**_**4**_^**–2**^, and **1-V**_**6**_**O**_**7**_**(OH)**_**6**_^**–2**^ for *x* = 0, 2, 4, and 6, respectively; Scheme 1).^17,18^ As depicted in Scheme 1, these organofunctionalized, hexavanadate assemblies mediate reversible 2H^+^/2e^–^ transfer reactions, resulting in access to reduced forms of the cluster featuring mixed valent vanadium cores and bridging hydroxide ligands.^15,17,18^ It is important to note that PCET reagents facilitate the transfer of multiple proton–electron pairs through a series of 1e^–^/1H^+^ steps; the measured BDFE(O–H) will be an average of the overall driving force for the dissociation of the individual bonds (BDFE(O–H)_avg_).^8,10^

The overall state of reduction of metal oxide compounds is known to impact the strength of surface bound H atoms.^17−21^ In an effort to gather further insight into the correlation between oxidation state and thermodynamics of PCET, we sought to establish the average strength of O–H bonds formed across the *entire* series of reduced **1-V**_**6**_**O**_**13**_^**–2**^ clusters, where BDFE(O–H)_avg_ is the average free energy to break two hydroxide bonds. We have reported BDFE(O–H)_avg_ values for 2e^–^/2H^+^ transfer from the most oxidized in this family of clusters [i.e. **1-V**_**6**_**O**_**11**_**(OH)**_**2**_^**–2**^, BDFE(O–H)_avg_ = 65.8 ± 0.1 kcal mol^–1^]^18^ and the most reduced [i.e., **1-V**_**6**_**O**_**7**_**(OH)**_**6**_^**–2**^, BDFE(O–H)_avg_ = 61.2 ± 0.4 kcal mol^–1^]^17^, rendering the determination of the average O–H bond strengths of **1-V**_**6**_**O**_**9**_**(OH)**_**4**_^**–2**^ (ox. state distrib. = V^IV^_4_V^V^_2_) necessary to complete the series.

**Figure 2:**
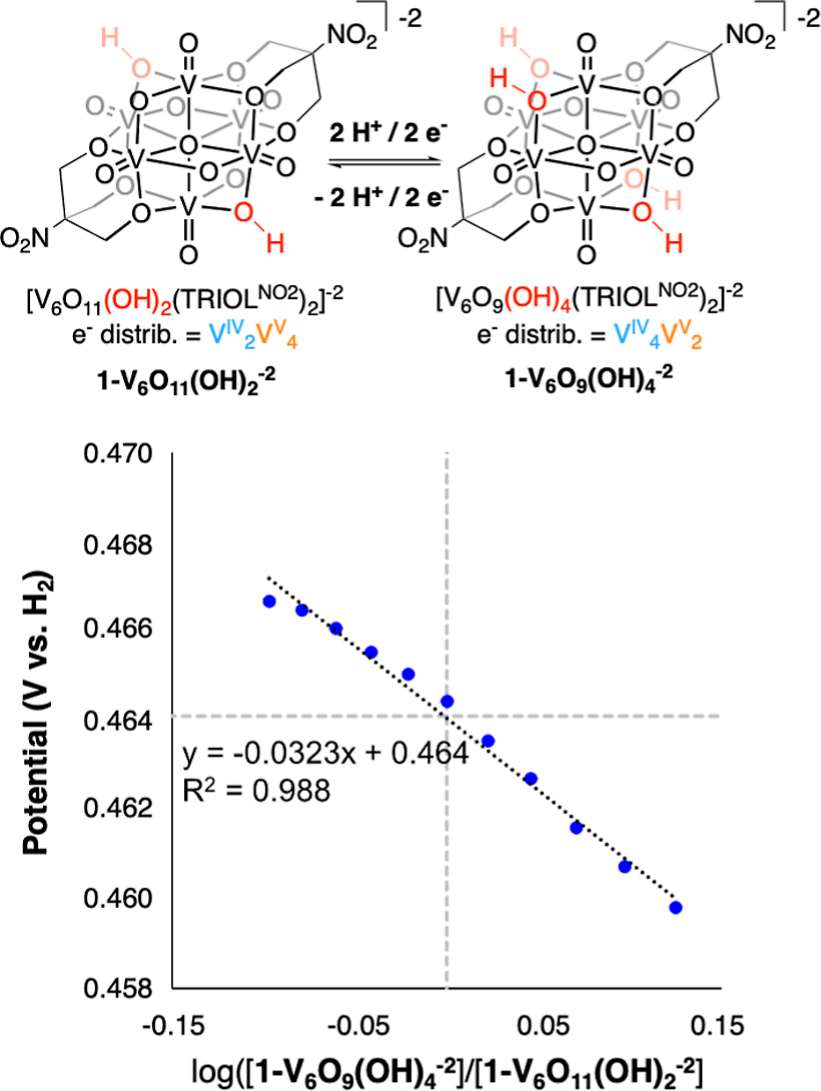
Scheme describing the H atom transfer reaction of relevance (top). Plot of the OCP value references against H_2_ measured at various ratios of **1-V**_**6**_**O**_**9**_**(OH)**_**4**_^**–2**^/**1-V**_**6**_**O**_**11**_**(OH)**_**2**_^**–2**^ against the log of the ratio of the concentrations of clusters (bottom). All measurements were performed in acetonitrile containing a 0.05 M buffer of 1:1 TEA/TEAH^+^ (p*K*_a_(TEAH^+^) = 18.8^24^) and supporting electrolyte (0.1 M [^n^Bu_4_N][PF_6_]). The slope of the line closely resembles the value expected by the Nernst equation for a 2H^+^/2e^–^ process. From the y-intercept (marked with dotted gray lines), the BDFE(O–H)_avg_ describing the loss of the first two H atom equivalents from the surface of **1-V**_**6**_**O**_**9**_**(OH)**_**4**_^**–2**^ is calculated using eq 2.

To measure the BDFE(O–H)_avg_ value describing the transfer of two H atom equivalents from the surface of **1-V**_**6**_**O**_**9**_**(OH)**_**4**_^**–2**^ to generate **1-V**_**6**_**O**_**11**_**(OH)**_**2**_^**–2**^, we turned to open circuit potential (OCP) analyses.^22,23^ By measuring the OCP of samples containing varying ratios of oxidized to reduced versions of the target compound, the potential required for H atom transfer can be directly obtained. The change in OCP as a function of the cluster concentration is shown in eq 1.
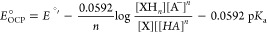
1where *E*_OCP_° is the measured OCP, *E*°^′^ is the standard potential, *n* represents the number of proton–electron pairs transferred, [XH_*n*_] and [X] represent the concentrations of the reduced and oxidized versions of the cluster, respectively, [HA] and [A^–^] are the concentrations of acid and conjugate base used as a buffer, respectively, and p*K*_a_ is the acid dissociation constant for acid used in the buffer. From these results, the BDFE(O–H)_avg_ can be calculated through the use of eq 2, in which the *E*_OCP_° is the OCP for a 1:1 ratio of oxidized to reduced cluster, and Δ*G*°[1/2*H*_2_(*g*)/H_1M_^•^] is a constant that accounts for the homolytic cleavage of H_2_ in the solvent of choice [Δ*G*°(1/2*H*_2_(*g*)/H_1M_^•^] = 52 kcal mol^–1^ in MeCN).^8^
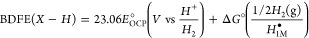
2We applied this experimental approach for the determination of the thermochemistry of the transfer of the first two H atom equivalents from **1-V**_**6**_**O**_**9**_**(OH)**_**4**_^**–2**^ to generate **1-V**_**6**_**O**_**11**_**(OH)**_**2**_^**–2**^ (Figures 2 and S6; Table 1). The resultant plots of potential vs the log of the ratio of reduced (**1-V**_**6**_**O**_**9**_**(OH)**_**4**_^**–2**^) to oxidized (**1-V**_**6**_**O**_**11**_**(OH)**_**2**_^**–2**^) clusters displayed a slope of −32.3 mV dec^–1^. This value is in good agreement with that predicted for a two proton two electron transfer event by the Nernst equation (29.6 mV dec^–1^), and with the results reported previously for **1-V**_**6**_**O**_**11**_**(OH)**_**2**_^**–2**^ (−37.7 ± 3.3 mV dec^–1^)^18^ and **1-V**_**6**_**O**_**7**_**(OH)**_**6**_^**–2**^ (−34.6 ± 0.004 mV dec^–1^).^17^ The potential required for the charge transfer to occur at **1-V**_**6**_**O**_**9**_**(OH)**_**4**_^**–2**^ can be obtained as the *y*-intercept of Figure 2 (0.464 V vs H^+^/H_2_); the BDFE(O–H)_avg_ is then calculated through the use of eq 2, resulting in a value of 62.6 ± 0.1 kcal mol^–1^ (Table 1). The BDFE(O–H)_avg_ of **1-V**_**6**_**O**_**9**_**(OH)**_**4**_^**–2**^ lies between those measured for **1-V**_**6**_**O**_**11**_**(OH)**_**2**_^**–2**^ (65.7 kcal mol^–1^)^18^ and **1-V**_**6**_**O**_**7**_**(OH)**_**6**_^**–2**^ (61.6 kcal mol^–1^),^17^ consistent with a decrease in O–H bond strengths with reduction of the metal oxide core.

Density functional theory (DFT) calculations were performed to corroborate and further scrutinize the H atom transfer reaction thermodynamics. The Gibbs free energy of each cluster was calculated at different reduction states. The corresponding BDFE(O–H)_avg_ were calculated per eq 3; individual BDFE(O–H) are provided in the Supporting Information (Table S3) per eq S1.
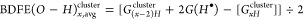
3

**Figure 3:**
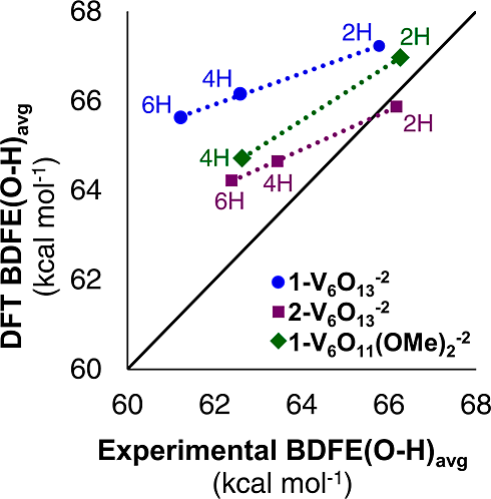
Parity plot between DFT calculated and experimentally determined BDFE(O–H)_avg_ values. Blue circles: [V_6_O_13–*x*_(OH)_*x*_(TRIOL^NO2^)_2_]^−2^ (**1-V**_**6**_**O**_**13**_^**–2**^); purple squares: [V_6_O_13–*x*_(OH)_*x*_(TRIOL^Me^)_2_]^−2^ (**2-V**_**6**_**O**_**13**_^**–2**^); green diamonds: [V_6_O_11–*x*_(OMe)_2_(OH)_*x*_(TRIOL^NO2^)_2_]^−2^ (**1-V**_**6**_**O**_**11**_**(OMe)**_**2**_^**–2**^). Here, “*x*” denotes the number of surface-adsorbed hydrogen atoms (*x* = 2, 4, 6).

In eq 3, *G*_*x*H_^cluster^ is the Gibbs free energy of the cluster possessing *x* surface (O–H) equivalents and *G*(*H*^•^) is the corresponding Gibbs free energy of the hydrogen atom. Using eq 3, we calculated the BDFE(O–H)_avg_ values for the nitro-backed POV-alkoxide clusters with *x* = 2 (**1-V**_**6**_**O**_**11**_**(OH)**_**2**_^**–2**^), 4 (**1-V**_**6**_**O**_**9**_**(OH)**_**4**_^**–2**^), and 6 (**1-V**_**6**_**O**_**7**_**(OH)**_**6**_^**–2**^) H atoms; the DFT predictions are plotted against the experimentally derived BDFE(O–H)_avg_ (from Table 1) in Figure 3.

**Table 1:** Tabulated Results From the OCP Experiments to Determine the BDFE(O–H)_avg_ for Each Cluster Reported^f^

cluster	electron distribution	buffer	OCP (V vs H_2_)	BDFE(O–H)_avg_ (kcal mol^–^^1^)	slope (mV/dec)
1-V_6_O_11_(OH)_2_^–^^2^	V^IV^_2_V^V^_4_	DMA/DMAH^+^^a^	0.597	65.8 ± 0.13	–35.1
1-V_6_O_9_(OH)_4_^–^^2^	V^IV^_4_V^V^_2_	TEA/TEAH^+^^b^	0.459	62.6 ± 0.11	–29.3
1-V_6_O_7_(OH)_6_^–^^2^	V^IV^_6_	TMG/TMGH^+^^c^	0.400	61.2 ± 0.35	–26.7
2-V_6_O_11_(OH)_2_^–^^2^	V^IV^_2_V^V^_4_	Pyrd/PyrdH^+^^d^	0.614	66.2 ± 0.14	–32.0
2-V_6_O_9_(OH)_4_^–^^2^	V^IV^_4_V^V^_2_	TEA/TEAH^+^^b^	0.496	63.4 ± 0.72	–29.9
2-V_6_O_7_(OH)_6_^–^^2^	V^IV^_6_	TMG/TMGH^+^^c^	0.450	62.4 ± 0.24	–32.1
1-V_6_O_9_(OMe)_2_(OH)_2_^–^^2^	V^IV^_4_V^V^_2_	ClPyrd/ClPyrdH^+^^e^	0.618	66.3 ± 0.49	–26.1
1-V_6_O_7_(OMe)_2_(OH)_4_^–^^2^	V^IV^_6_	Pyrd/PyrdH^+^^d^	0.461	62.6 ± 0.65	–28.1

a DMA = *N*,*N*-dimethylaniline; DMAH^+^ = *N*,*N*-dimethylanilinium tetrafluoroborate.

b TEA = triethylamine; TEAH^+^ = triethylammonium tetrafluoroborate.

c TMG = 1,1,3,3-tetramethylguanidine; TMGH^+^ = 1,1,3,3-tetramethylguanidinium tetrafluoroborate.

d Pyrd = pyridine; PyrdH^+^ = pyridinium tetrafluoroborate.

e ClPyrd = 2-chloropyridine; ClPyrdH^+^ = 2-chloropyridinium tetrafluoroborate.

f For clarity, we have included the number of reduced vanadium centers, the buffer identity, the measured OCP value, and the slope of the linear best fit line.

For the redox series of [V_6_O_13–*x*_(OH)_*x*_(TRIOL^NO2^)_2_]^−2^ clusters, calculations support the decrease in BDFE(O–H)_avg_ values with increasing degree of hydrogenation (and corresponding reduction of the cluster V centers; per Table 1 above). This is in agreement with the experimental trends (the per-cluster trends align essentially parallel with the parity line). For each step change of 2H atom equivalents, both the DFT and experimental BDFE(O–H)_avg_ change by approximately the same amount.

The small offset between experimental and computational parity of the BDFE(O–H)_avg_ values for [V_6_O_13–*x*_(OH)_*x*_(TRIOL^NO2^)_2_]^−2^ shown in Figure 3 can be attributed to the complex solvation environment in experiments that is not captured in the DFT calculations (see Computational Methods section). These include, among others, the presence of explicit solvent molecules, the presence of ions from buffers and the electrolyte, electrode surface effects, etc. We note that this computational treatment is relevant in all clusters studied in this work. Thus, the DFT calculations can independently reproduce the experimentally derived trends in BDFE(O–H)_avg_ values for the POV-alkoxide clusters to a very satisfactory degree given the complexity of the solution environment in experiments.

The general trend in BDFE(O–H)_avg_ value as a function of the oxidation state distribution of vanadium ions composing the POV-alkoxide core is consistent with precedent in molecules and materials. Several examples of mononuclear inorganic complexes with the ability to perform multielectron-multiproton transfer chemistry have been shown to form more reactive O–H bonds as hydrogen atoms are added to the complex.^25−31^ Likely the most relevant comparison between the observed changes in BDFE(O–H)_avg_ in our clusters is noted in the work of the Mayer group with CeO_2_ nanoparticles. In a series of publications, Mayer and co-workers analyze the thermochemical trends of H atom transfers across a range of states of reduction for colloidal CeO_2_ nanoparticles.^24,32^ The authors determine that the extent of reduction at the metal oxide assembly directly impacts the strength of surface bound hydroxide ligands,^24^ consistent with the trends described above for the POV-alkoxide clusters. The authors highlight the fact that the differences in BDFE(O–H) as a function of state of reduction of the nanoparticle disagrees with values predicted by the Nernst equation; the experimental BDFE(O–H) values measured are approximately 20 times larger than those calculated. While the authors were unable to conclusively establish the reasoning behind this observation, they propose that deviation from an ideal delocalized electronic structure in the CeO_2_ nanoparticles is responsible for the observed differences in calculated and measured BDFE(O–H).

Further evidence to support the theory of localization of charge density at metal oxide surface imparting a significant impact on the magnitude of BDFE(O–H) values can be found in experimental results from Christou and co-workers.^33^ Crystallographic analysis of the structure of cerium oxide clusters reveals distortions in the Ce–O bond lengths depending on oxidation state distribution of metal centers. This indicates that the charge density at the metal oxide core is not delocalized across the entire metal oxide structure, instead concentrated at distinct, surface metal ions.^33^ The differences in oxidation state of the metal centers results in variations in the ligation preferences at the surface of the material, where the oxidized Ce sites are capable of forming more thermodynamically stable hydroxide.

Natural population analysis computations make plain the existence and degree of localization of the reducing electron density, with regard to the charge distributed on the bridging oxygens and adjacent vanadium sites upon hydrogen reduction (precise values are provided in the Supporting Information: Figure S28, Tables S5–S7). For example, upon addition of 2H atom equivalents to **1-V**_**6**_**O**_**13**_^**–2**^, one observes that for each H atom added, electron density is distributed across the cluster such that it primarily affects the local charge of the protonated bridging oxygens and secondarily of the vanadium centers. The charges of the remaining bridging oxygens are only minorly affected. This results in significant heterogeneity of the charge distribution on the bridging oxygens, which are the sites involved in the hydrogen transfer processes in the BDFE(O–H). This observation rationalizes the differences in the ΔBDFE(O–H)_avg_ between experiments and what is expected from the Nernst equation. We note that these degrees of charge localization are approximately consistent through all three reduced clusters [i.e., **1-V**_**6**_**O**_**11**_**(OH)**_**2**_^**–2**^, **1-V**_**6**_**O**_**9**_**(OH)**_**4**_^**–2**^, and **1-V**_**6**_**O**_**7**_**(OH)**_**6**_^**–2**^]. Interestingly, one notes that the bridging oxygen charge heterogeneity is a feature of the clusters with mixed V valences, i.e. **1-V**_**6**_**O**_**11**_**(OH)**_**2**_^**–2**^ and **1-V**_**6**_**O**_**9**_**(OH)**_**4**_^**–2**^, and that they depart more from idealized electronically delocalized structures as compared to **1-V**_**6**_**O**_**13**_^**–2**^ and **1-V**_**6**_**O**_**7**_**(OH)**_**6**_^**–2**^.

### Effect of Ligand Modifications on the BDFE(O–H)_avg_ of Reduced POV-Alkoxide Clusters

Prior work by Zubieta and co-workers describes significant changes in the redox properties of POV-alkoxide clusters, namely [V_6_O_13_(TRIOL^R^)_2_]^2–^ (TRIOL = tris (hydroxymethyl)methane; R = Me, Et, CH_2_OH, NO_2_, NMe_2_, and Bn), upon modification of the peripheral “R” group of the trisalkoxymethane (TRIOL^R^) ligands.^15^ Changing the peripheral “R” substituent of the POV-alkoxide cluster from a nitro group to a methyl moiety results in a cathodic shift of reduction potentials of the cluster core [*E*_1/2_ values of **1-V**_**6**_**O**_**13**_^**–2**^: [V_6_O_13_(TRIOL^NO2^)_2_]^−2/–3^ = −0.62 V; [V_6_O_13_(TRIOL^NO2^)_2_]^−3/–4^ = −1.42 V vs Fc^+/0^; *E*_1/2_ values of **2-V**_**6**_**O**_**13**_^**–2**^**:** [V_6_O_13_(TRIOL^Me^)_2_]^−2/–3^ = −0.92 V; [V_6_O_13_(TRIOL^Me^)_2_]^−3/–4^ = −1.69 V vs Fc^+/0^ in MeCN].^15,16^ This observation is consistent with the increased electron donating character of methyl groups in comparison to nitro substituents, which in turn increases the electron density at the vanadium oxide core. The observed shift in reduction potential led us to consider whether the BDFE(O–H)_avg_ of the surface hydroxide ligands formed upon reduction of these clusters might likewise be affected.

**Figure 4:**
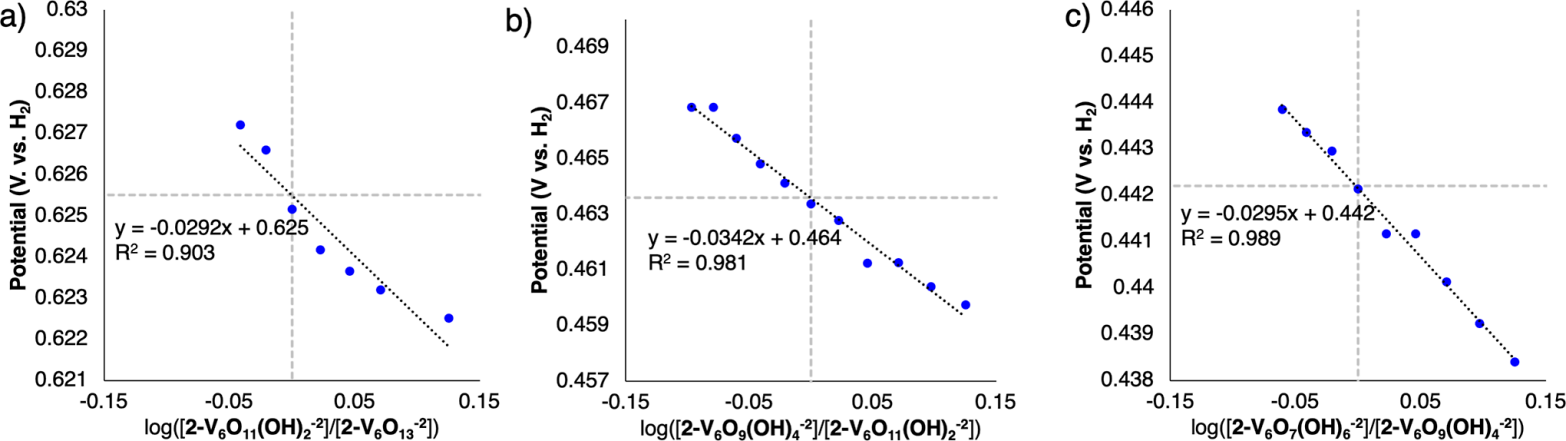
Plots of the resulting OCP values to obtain the BDFE(O–H)_avg_ for (A) **2-V**_**6**_**O**_**11**_**(OH)**_**2**_^**–2**^; (B) **2-V**_**6**_**O**_**9**_**(OH)**_**4**_^**–2**^; and (C) **2-V**_**6**_**O**_**7**_**(OH)**_**6**_^**–2**^. Each experiment is run in acetonitrile with 0.1 M [^n^Bu_4_N][PF_6_] as supporting electrolyte and 50 mM of a buffer containing a 1:1 mixture an organic acid and conjugate base pair (identity of each buffer can be found in Table 1). Each potential collected and initially referenced to Fc^+/0^, where the value is then converted to be referenced to the H^+^/H_2_ potential in acetonitrile.^23^

Initial experiments focused on a derivative of the high-valent POV-alkoxide cluster featuring a methyl group on the TRIOL ligand in place of the nitro (i.e., [V_6_O_13_(TRIOL^Me^)_2_]^2–^ (**2-V**_**6**_**O**_**13**_^**–2**^; TRIOL^Me^ = 1,1,1-trishydroxymethylethane). Zubieta has reported the isolation of the 4e^–^/4H^+^ reduced compound [V_6_O_9_(OH)_4_(TRIOL^Me^)_2_]^−2^ (**2-V**_**6**_**O**_**9**_**(OH)**_**4**_^**–2**^), and the 6e^–^/6H^+^ reduced species, [V_6_O_7_(OH)_6_(TRIOL^Me^)_2_]^−2^ (**2-V**_**6**_**O**_**7**_**(OH)**_**6**_^**–2**^).^15^ To determine whether trends in relative BDFE(O–H)_avg_ of surface hydroxide moieties in reduced POV-alkoxide clusters remain consistent after altering the identity of the tridentate ligand, we performed a series of OCP analyses in order to measure BDFE(O–H)_avg_ values for the series of reduced clusters, including: [V_6_O_11_(OH)_2_(TRIOL^Me^)_2_]^−2^ (**2-V**_**6**_**O**_**11**_**(OH)**_**2**_^**–2**^; BDFE(O–H)_avg_ = 66.2 ± 0.14 kcal mol^−1^), **2-V**_**6**_**O**_**9**_**(OH)**_**4**_^**–2**^ (BDFE(O–H)_avg_ = 63.4 ± 0.72 kcal mol^−1^), and **2-V**_**6**_**O**_**7**_**(OH)**_**6**_^**–2**^ (BDFE(O–H)_avg_ = 62.4 ± 0.24 kcal mol^−1^; Table 1, Figure 4 and Figures S8–S10).

Inspection of the measured BDFE(O–H)_avg_ values for complexes **2-V**_**6**_**O**_**11**_**(OH)**_**2**_^**–2**^, **2-V**_**6**_**O**_**9**_**(OH)**_**4**_^**–2**^, and **2-V**_**6**_**O**_**7**_**(OH)**_**6**_^**–2**^ reveals that the methyl- and nitro-backed POV-alkoxide clusters possess similar thermochemistry of surface O–H moieties (Table 1). The small extent to which the capping tridentate ligand influences the energy required for H atom uptake initially seems counterintuitive given that the TRIOL^R^ ligand is able to significantly influence the redox properties of the metal oxide core.^15,16^ Consistent with our prior observation that increased reduction of the assembly weakens the strength of surface O–H bonds, we would expect that the increased electron density at the cluster core imparted by the electron donating methyl-backed TRIOL ligands should result in a lower affinity of H atoms for the surface of the POV-alkoxide. We hypothesize that the small degree of variation of BDFE(O–H)_avg_ values across the methyl- and nitro- backed POV-alkoxide clusters is due to the fact that changes in the redox characteristics of the POV-alkoxide cluster are paired with proportional changes to the basicity of the surface of the assembly (Figure S14). The offsetting changes in free energy, typically referred to as thermodynamic compensation, results in little to no difference in the BDFE. Similar observations have been made in examples of mononuclear inorganic complexes, wherein the change in free energy of reduction is offset by the change in free energy required for protonation, resulting in little to no change in the overall energy required for transfer of an H atom.^8,34,35^

**Figure 5:**
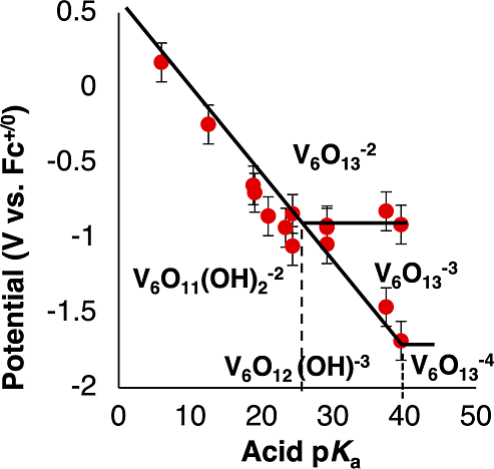
Potential-p*K*_a_ diagram for **2-V**_**6**_**O**_**13**_^**–2**^. Red data points represent the measured reduction potential of 1 mM **2-V**_**6**_**O**_**13**_^**–2**^ in acetonitrile with supporting electrolyte (0.1 M [^n^Bu_4_N][PF_6_]) plotted against the p*K*_a_ of the various organic acids (2 mM) in solution. The horizontal black lines represent p*K*_a_ independent redox events and the diagonal lines represent p*K*_a_ dependent redox events with a slope of 59.4 mV/dec corresponding to a 1:1 proton coupled event. See the Supporting Information for more information, and for the cyclic voltammogram associated with each data point (Figures S15–S24). Each region of the diagram is labeled with the most stable species at the associated potential and p*K*_a_.

To experimentally confirm the increased surface basicity of the methyl substituted cluster, **2-V**_**6**_**O**_**13**_^**–2**^, we constructed a potential-p*K*_a_ diagram.^36^ CVs of 1.0 mM cluster were measured in the presence of 2.0 mM organic acid (p*K*_a_ 5.6–39.5); due to the complicated nature of these cyclic voltammograms, the half wave potentials were determined by examining the second derivative of each CV according to a method reported by Vullev.^37^ The *E*_1/2_ values of the resulting redox events were plotted against the p*K*_a_ of the acid to generate the plot in Figures 5 and S15–S24.

Using the Bordwell equation and our experimentally determined BDFE(O–H)_avg_ (66.2 kcal mol^–1^), we generated a linear regression with the Nernstian slope of 59.4 mV/dec (see Supporting Information for more detail). This line fits within error of the experimental plot thereby confirming the BDFE(O–H)_avg_ value obtained from OCP measurements. We can approximate p*K*_a_ values for the two hydrogens bound to **2-V**_**6**_**O**_**11**_**(OH)**_**2**_^**–2**^ as the points where the acid independent regions intersect with the acid dependent regions (marked with dotted lines). p*K*_a_ values of 28.4 and 40.2 are assigned as the dissociation constants for the first and second proton removed from the surface of the reduced cluster. These values indicate that the cluster surface is significantly more basic than its nitro substituted counterpart (p*K*_a_s 19.3 and 32.7, respectively).

**Figure 6:**
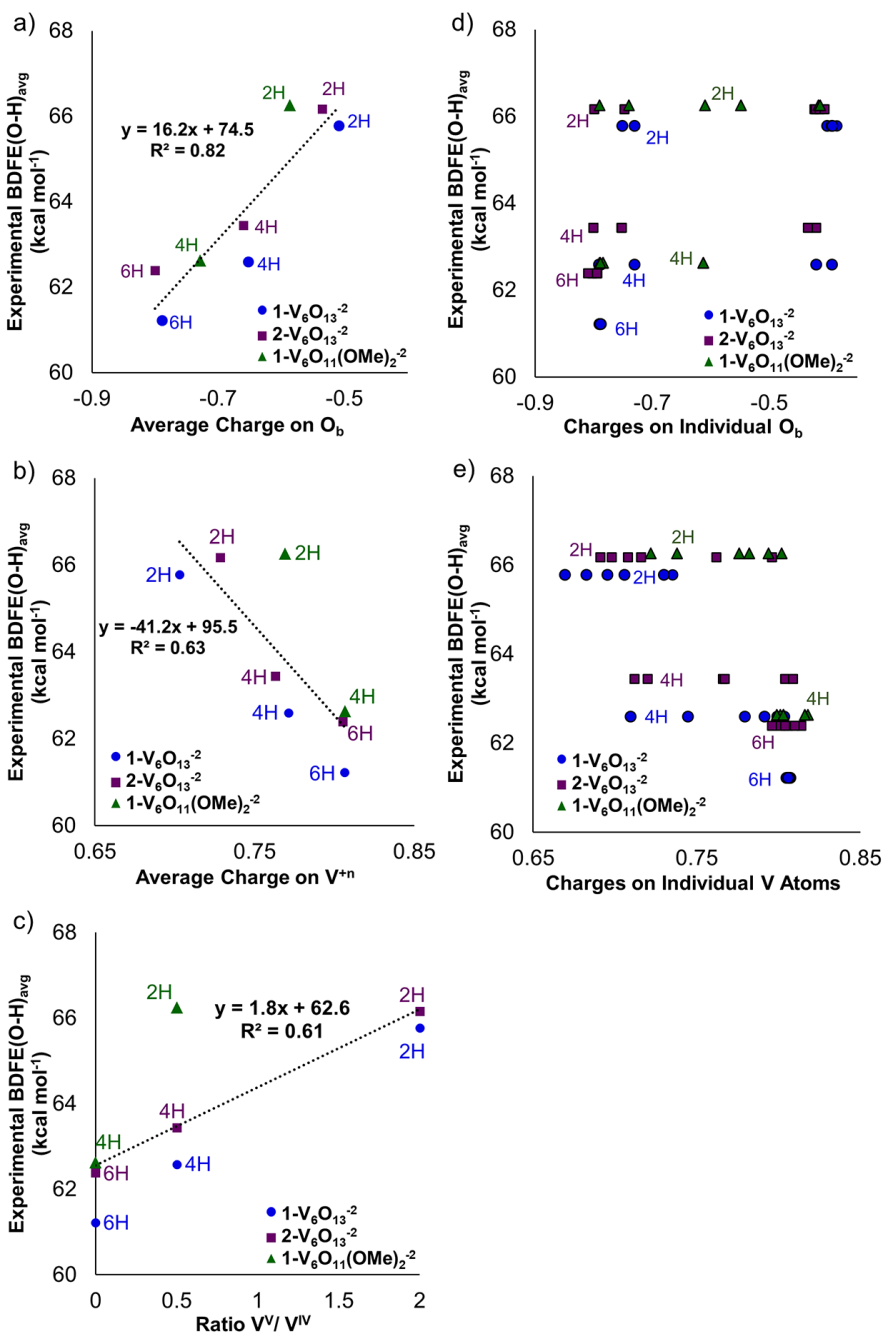
Experimentally measured BDFE(O–H)_avg_ vs DFT-calculated charge distribution on the (a) bridging oxygen and (b) vanadium atoms of the clusters and (c) ratio of number of V atoms at different oxidation states (i.e., V^V^/V^IV^). Panels (d,e) show the BDFE(O–H)_avg_ vs the individual bridging oxygen and vanadium atomic charges, respectively. The DFT charges are calculated on the specific *x* = 2, 4, 6 clusters (e.g; not from the average of values for *x* = 1 and *x* = 2). Blue circles: [V_6_O_13–*x*_(OH)_*x*_(TRIOL^NO2^)_2_]^−2^. Purple squares: [V_6_O_13–*x*_(OH)_*x*_(TRIOL^Me^)_2_]^−2^. Green diamonds: [V_6_O_11–*x*_(OMe)_2_(OH)_*x*_(TRIOL^NO2^)_2_]^−2^.

Computationally, we further explore the effect of cluster charge distribution on BDFE(O–H)_avg_ and how they are affected by the ligand environment (see reported atomic charges in Figure S28 and Tables S5–S7). We expect that varying the charge distributed to the bridging oxygens changes their basicity, which is consistent with the BDFE(O–H)_avg_ of the formed hydroxyl groups being significantly impacted with the degree of cluster hydrogenation. Herein, the varying charge distribution to the bridging oxygens (i.e., relative increases or decreases in localized electron density) can be affected by either the degree of hydrogenation or changes to the TRIOL ligands (and their electron donating or withdrawing characteristics). This is supported by Figure 6, where we plot the experimental BDFE(O–H)_avg_ vs the DFT calculated charge distribution on bridging oxygen and vanadium sites, and the V oxidation states (ratio of V^V^/V^IV^). Figure 6a show that all experimentally measured BDFE(O–H)_avg_ of the different clusters at various degrees of reduction correlate with the charge localized on the bridging oxygens. This observation agrees with recent computational and experimental studies on H_*x*_WO_3_ by Miu et al.,^38,39^ where intercalation of hydrogen atoms into the bulk changed the electronic properties of the oxide, including the basicity of the surface oxygen atoms, which is a descriptor for the interaction of hydrogen atoms with the oxide’s surface oxygens. Figure 6b shows, relative to Figure 6a, that the BDFE(O–H)_avg_ is strongly correlated to the charge of the bridging oxygens, but less to that of the vanadium centers. Figure 6d,e further illustrate that in mixed valence clusters, there is greater charge variability among the bridging oxygens and less so for the vanadium centers. This is consistent with the charge transfer from the reducing H atoms being localized primarily on the bridging oxygens. It should be noticed that neither the charge on the V sites (Figure 6b), nor the V oxidation state changes (calculated as the ratio of V atoms with different oxidation states in Figure 6c) can effectively describe the BDFE(O–H)_avg_ changes between the different clusters. The DFT charge distribution data also support the experimentally observed increase in basicity. This is demonstrated in Figure 6a, where the DFT-calculated charge distribution on the bridging oxygens is uniformly higher (purple points are shifted to more negative charge values compared to blue data) in the methyl-backed POV-alkoxide clusters compared to the nitro-backed clusters.

We further computationally dissect the BDFE(O–H)_avg_ into their electron transfer and proton transfer energies via construction of a thermodynamic Square Scheme (Figure S27a).^8,40^ The energies of each side of the square scheme may be calculated and are presented accordingly in Figure S27c–f. One first notes the high magnitudes of both the electron and proton transfers; this observation validates that these systems operate via concerted proton–electron transfer. The proton and electron transfer energetics, both between the different cluster series and across their degrees of reduction, may also be compared (Figure S27). It is observed that where electron transfer becomes less exothermic from one cluster to another (i.e., decrease in cluster reducibility), the accompanying proton transfer to the cluster becomes less endothermic (i.e., the cluster becomes more basic). This confirms the experimentally observed thermodynamic compensation effect.

### Direct Alkylation of the POV-Alkoxide Surface

**Scheme 2:**
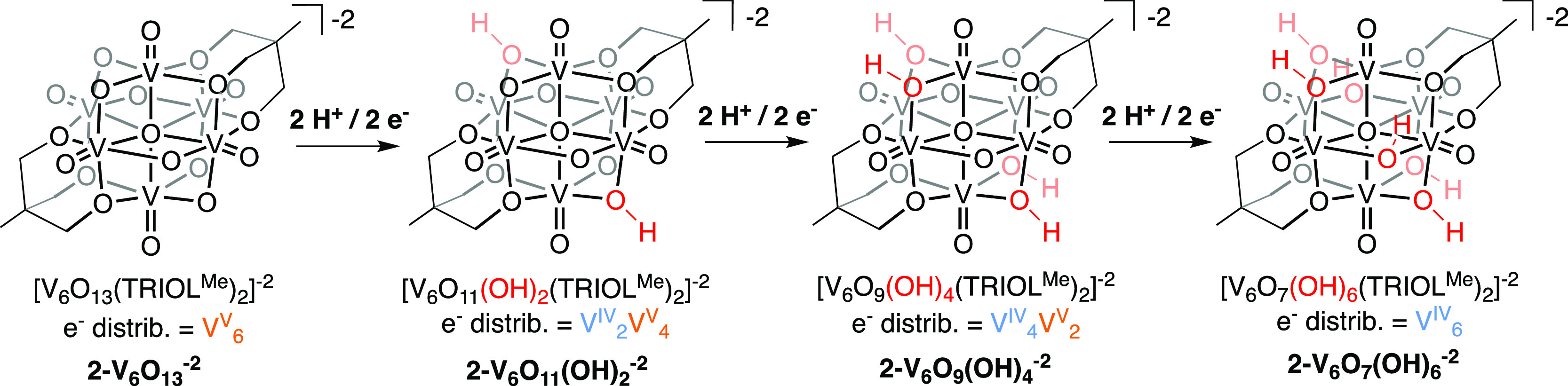
H-Atom Uptake at **2-V**_**6**_**O**_**13**_^**–2**^; Access to Reduced Forms of the Lindqvist-Type POV-Alkoxide Cluster via Proton-Coupled Electron Transfer

The aforementioned experiments indicate that modifications to the peripheral tridentate ligands do not influence the thermodynamics of H atom uptake at POV-alkoxide clusters. This finding inspired our team to probe alternative synthetic methods that would alter the affinity of the cluster surface for H atom equivalents. Based on our observations above that partial delocalization of charge greatly influences the magnitude of ΔBDFE(O–H), we hypothesized that synthetic modifications that further disrupt electronic communication across the metal oxide core would translate to distinct thermodynamics for H atom uptake at the cluster surface.^20^ Indeed, direct alkylation has been shown to reduce the symmetry of the POV-alkoxide cluster, and electron delocalization across the Lindqvist ion. In previous work, part of our team was able to effectively alkylate the surface of the POV-alkoxide cluster through the addition of either one or two equivalents of methylating reagent, resulting in the formation of [V_6_O_12_(OMe)(TRIOL^R^)_2_]^−1^ and [V_6_O_11_(OMe)_2_(TRIOL^R^)_2_] (R = NO_2_, Me). Here, we elected to focus our attention on the nitro-backed clusters containing two methoxide ligands in order to directly compare BDFE(O–H)_avg_ values with the 2e^–^/2H^+^ processes measured for the unfunctionalized clusters Scheme 2.

**Scheme 3:**
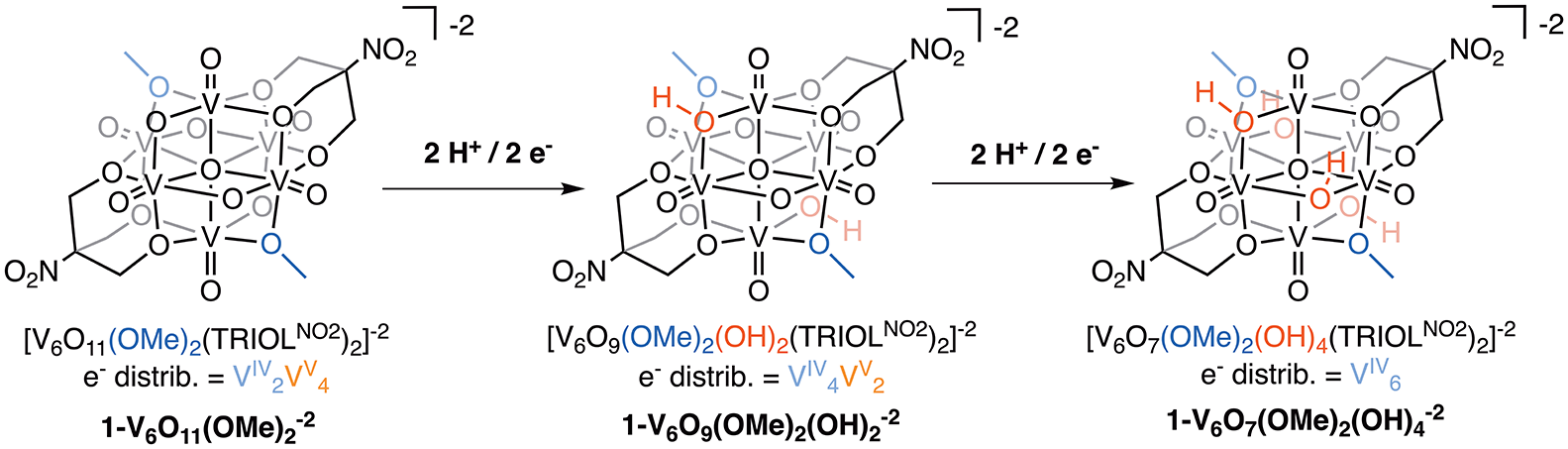
Synthesis of 1-V_6_O_9_(OMe)_2_(OH)_2_^–2^ and 1-V_6_O_7_(OMe)_2_(OH)_4_^–2^

The syntheses of reduced forms of the bis-methylated cluster, [V_6_O_11_(OMe)_2_(TRIOL^NO2^)_2_]^–2^ (**1-V**_**6**_**O**_**11**_**(OMe)**_**2**_^**–2**^), namely [V_6_O_9_(OMe)_2_(OH)_2_(TRIOL^NO2^)_2_]^−2^ (**1-V**_**6**_**O**_**9**_**(OMe)**_**2**_**(OH)**_**2**_^**–2**^), and [V_6_O_7_(OMe)_2_(OH)_4_(TRIOL^NO2^)_2_]^−2^ (**1-V**_**6**_**O**_**7**_**(OMe)**_**2**_**(OH)**_**4**_^**–2**^), were performed via addition of stoichiometric amounts of hydrazobenzene (e.g., 1 or 2 equiv) to a DCM solution containing the parent cluster, **1-V**_**6**_**O**_**11**_**(OMe)**_**2**_^**–2**^ (Scheme 3). In both cases, the reaction was stirred for several hours to ensure conversion to the reduced product. The products were isolated following a brief workup, resulting in the isolation of **1-V**_**6**_**O**_**9**_**(OMe)**_**2**_**(OH)**_**2**_^**–2**^ as a blue solid and **1-V**_**6**_**O**_**7**_**(OMe)**_**2**_**(OH)**_**4**_^**–2**^ as a light green powder (see Experimental and Computational Methods section for additional detail). Confirmation of the formation of the reduced clusters was performed via ESI–MS; peaks centered on the desired *m*/*z* value ([V_6_O_9_(OMe)_2_(OH)_2_]^−2^ = 421.2 *m*/*z*, [V_6_O_7_(OMe)_2_(OH)_4_]^−2^ = 421.8 *m*/*z*) were observed (Figures S3–S4).

**Figure 7:**
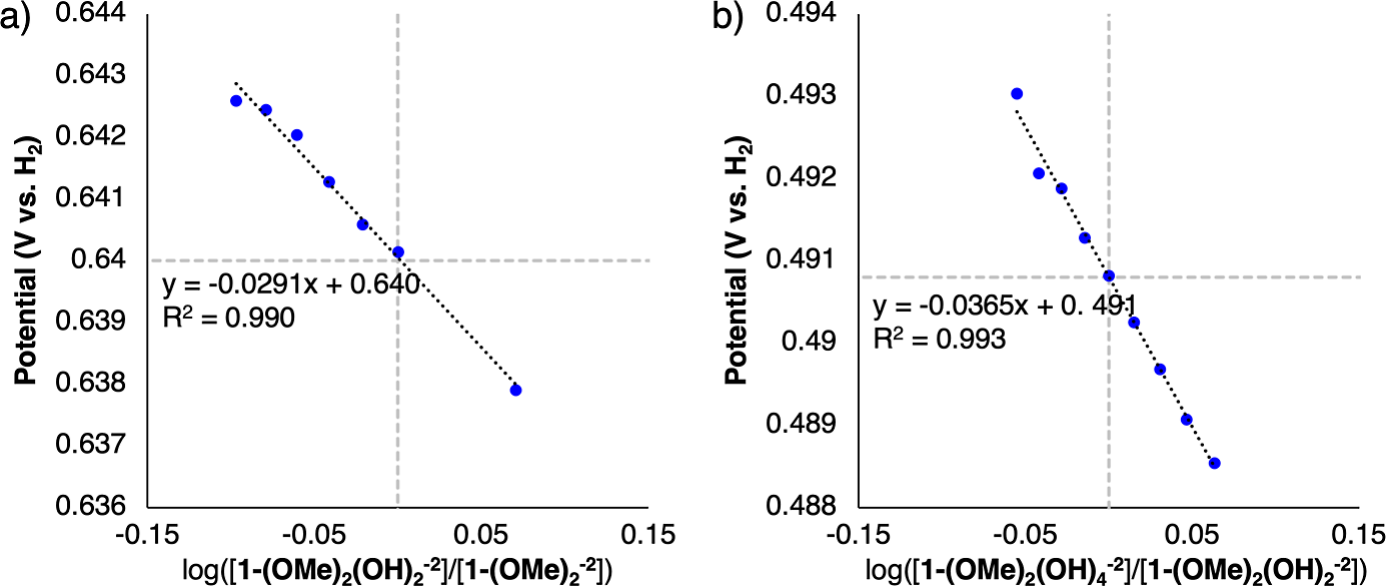
Plots of the resulting OCP values to obtain the BDFE(O–H)_avg_ for (a) **1-V**_**6**_**O**_**9**_**(OMe)**_**2**_**(OH)**_**2**_^**–2**^; (b) **1-V**_**6**_**O**_**7**_**(OMe)**_**2**_**(OH)**_**4**_^**–2**^. Each experiment is run in acetonitrile with 0.1 M [^*n*^Bu_4_N][PF_6_] as supporting electrolyte and 50 mM of a buffer containing a 1:1 mixture an organic acid and conjugate base pair (identity of each buffer can be found in Table 1). Each potential collected and initially referenced to Fc^+/0^, where the value is then converted to be referenced to the H^+^/H_2_ potential in acetonitrile, according to the literature procedure.^23^

With both reduced versions of the cluster isolated, we turned our attention to measuring the BDFE(O–H)_avg_ of **1-V**_**6**_**O**_**9**_**(OMe)**_**2**_**(OH)**_**2**_^**–2**^ and **1-V**_**6**_**O**_**7**_**(OMe)**_**2**_**(OH)**_**4**_^**–2**^, once again using the OCP analytical approach. Results of these experiments can be found in Figures 7 and S11–12 and in Table 1.

Comparison of the measured BDFE(O–H)_avg_ values for complexes **1-V**_**6**_**O**_**9**_**(OMe)**_**2**_**(OH)**_**2**_^**–2**^ and **1-V**_**6**_**O**_**7**_**(OMe)**_**2**_**(OH)**_**4**_^**–2**^ reveal a similar dependence of O–H bond strength on the oxidation state distribution of vanadium oxide ions contained within the core of the assembly. Specifically, the more reduced cluster, **1-V**_**6**_**O**_**7**_**(OMe)**_**2**_**(OH)**_**4**_^**–2**^ possesses average O–H bond strengths that are weaker than that of **1-V**_**6**_**O**_**9**_**(OMe)**_**2**_**(OH)**_**2**_^**–2**^; (BDFE(O–H)_avg_**1-V**_**6**_**O**_**9**_**(OMe)**_**2**_**(OH)**_**2**_^**–2**^ = 66.3 ± 0.49 kcal mol^–1^; BDFE(O–H)_avg_**1-V**_**6**_**O**_**7**_**(OMe)**_**2**_**(OH)**_**4**_^**–2**^ = 62.6 ± 0.65 kcal mol^–1^). The difference in the BDFE(O–H)_avg_ between the two variants of the bis-methylated clusters spans ∼4 kcal mol^–1^ representing a larger change in the thermodynamics of the surface O–H bonds than observed in the series of unfunctionalized clusters. The large ΔBDFE(O–H)_avg_ upon change in oxidation state for the alkylated POV-alkoxide cluster suggests that the addition of methyl groups to the surface of the assembly induces a change in electronic structure that limits delocalization of electron density. This, in turn, increases the sensitivity of the cluster surface to changes in oxidation states of vanadium ions.

The BDFE(O–H)_avg_ values of the methyl-substituted POV-alkoxide clusters can be compared to their isovalent unfunctionalized congeners (*e.g*. V^IV^_4_V^V^_2_O_9_(OH)_4_ and V^IV^_4_V^V^_2_O_9_ (OMe)_2_(OH)_2_), revealing an overall increase in BDFE(OH)_avg_ (ΔBDFE(O–H)_avg_ = 3.7 kcal mol^–1^) for the more oxidized variant, yet a surprisingly small change in BDFE(OH)_avg_ for the more reduced versions [i.e., **1-V**_**6**_**O**_**7**_**(OMe)**_**2**_**(OH)**_**4**_^**–2**^, BDFE(O–H) _avg_ = 62.6 ± 0.65 kcal mol^–1^ vs **1-V**_**6**_**O**_**7**_**(OH)**_**6**_^**–2**^, BDFE(O–H)_avg_ = 61.2 ± 0.35 kcal mol^–1^; ΔBDFE(O–H)_avg_ = 1.4 kcal mol^–1^]. To justify these trends more precisely, we turn our attention to the electrochemical characteristics of these clusters through the use of cyclic voltammetry. In particular, we were interested in the difference in the redox potentials of adjacent events, as this would enable us to define the extent of delocalization of charge density across the metal oxide core. The *K*_c_ of **1-V**_**6**_**O**_**11**_**(OMe)**_**2**_^**–2**^ has been reported by our group previously, where the difference in the reduction events of the cluster reveal a *K*_c_ value of 9.8 × 10^9^.^16^ This value is significantly smaller than that for **1-V**_**6**_**O**_**13**_^**–2**^ (*K*_c_ = 5.3 × 10^13^), indicating that addition of the methyl groups to the surface of the cluster results in a decrease in the ability for the vanadium centers to electronically communicate.^20^ This increase in localization of charge density can account for not only the differences in the BDFE(O–H)_avg_ between the functionalized and unfunctionalized versions of the clusters but also the variations in the ΔBDFE(O–H)_avg_ across various oxidation states.

The impact of vanadium oxidation state and electronic communication is again highlighted in Figure 6 by noting the differing alignment of **1-V**_**6**_**O**_**9**_**(OMe)**_**2**_**(OH)**_**2**_^**–2**^ vs the other clusters (Figure 6b,c). Being reduced by the two surface methyl groups, its average vanadium charge is similar to that of **1-V**_**6**_**O**_**9**_**(OH)**_**4**_^**–2**^ and **2-V**_**6**_**O**_**9**_**(OH)**_**4**_^**–2**^ (Figure 6b), all three of which have the same vanadium oxidation state distribution (Figure 6c), but the BDFE(O–H)_avg_ of **1-V**_**6**_**O**_**9**_**(OMe)**_**2**_**(OH)**_**2**_^**–2**^ is closer in value to clusters that possess similar extents of hydrogenation [e.g., **1-V**_**6**_**O**_**11**_**(OH)**_**2**_^**–2**^ and **2-V**_**6**_**O**_**11**_**(OH)**_**2**_^**–2**^]. Additionally, the fact that the experimental results showed the smallest BDFE(O–H)_avg_ deviation in the more reduced clusters and the largest deviations in the partially reduced ones can be potentially explained by the oxidation states of V atoms and the electron density localization (see Tables S5−S7). In the case of the fully reduced, isovalent (e.g., V^IV^_6_) POV-alkoxides (**1-V**_**6**_**O**_**7**_**(OMe)**_**2**_**(OH)**_**4**_^**–2**^ and **1-V**_**6**_**O**_**7**_**(OH)**_**6**_^**–2**^), since all vanadium centers are in the V^IV^ oxidation state and all bridging oxygens are coordinated with hydrogen atoms or methyl substituents, the electron density and the charge distribution on the cluster becomes more homogeneous, as illustrated in Figure 6d,e. This likely explains the smaller deviation in the absolute values of BDFE(O–H)_avg_ between these specific clusters. In contrast, in the examples of mixed valent POV-alkoxide clusters, complexes **1-V**_**6**_**O**_**9**_**(OMe)**_**2**_**(OH)**_**2**_^**–2**^ and **1-V**_**6**_**O**_**9**_**(OH)**_**4**_^**–2**^, there is a charge localization on specific centers of the clusters due to the fact that the bridging oxygens are only partially coordinated (i.e., 4 out of 6 oxygens are coordinated) and the vanadium atoms possess mixed valences (Figure 6c). The differences in localization and heterogeneities between these two clusters correspond to decreased charge density in the average bridging oxygens for **1-V**_**6**_**O**_**9**_**(OMe)**_**2**_**(OH)**_**2**_^**–2**^ and increased BDFE(O–H)_avg_ relative to **1-V**_**6**_**O**_**9**_**(OH)**_**4**_^**–2**^. Thus, in this specific case with mixed vanadium oxidation states, the presence of methyl ligands disturbs the charge distribution on the cluster dramatically affecting the BDFE(O–H)_avg_ values. We highlight again that the localized charge on the bridging oxygens effectively describes the experimentally observed BDFE(O–H)_avg_ changes (Figure 6a,d) despite the fact that the surface functionalization and the degree of hydrogenation of the different clusters result in V atoms with mixed oxidation states and charge distributions (Figure 6c,e,b).

## Conclusions

Here, we present the detailed thermodynamics of H atom transfer of 2H^+^/2e^–^ reduced POV-alkoxide clusters by combining synthesis, characterization, and electrochemical experiments as well as DFT calculations. We first investigated the V_6_O_13-x_(OH)_*x*_(TRIOL^R^)_2_^–2^ clusters, which feature 2, 4, and 6 reactive protons bound to bridging oxide ligands at the molecule’s surface. In addition, we modified organic ligands at the surface of the assembly with electron donating/withdrawing functional groups at both peripheral TRIOL capping sites. BDFE(O–H)_avg_ values of the resulting surface hydroxides are reported. As is consistent with the literature for nanomaterials, reduction of the metal oxide cluster produces more reactive H atoms at its surface. Importantly, the partial localization of electron density within the cluster core results in a much larger range of BDFE(O–H)_avg_ values than predicted by the Nernst equation, spanning 4.6 kcal mol^–1^ [**1-V**_**6**_**O**_**11**_**(OH)**_**2**_^**–2**^, 65.8 kcal mol^–1^; **1-V**_**6**_**O**_**9**_**(OH)**_**4**_^**–2**^, 62.6 kcal mol^–1^; and **1-V**_**6**_**O**_**7**_**(OH)**_**6**_^**–2**^, 61.2 kcal mol^–1^]. The same trend in BDFE(O–H)_avg_ values is observed when the nitro group on the peripheral TRIOL ligand is replaced by a methyl group. Despite the significant negative shift in reduction potential of this cluster (∼0.25 V), there is only about a 1 kcal mol^–1^ change in BDFE(O–H)_avg_ values for nitro and methyl clusters at the same extent of oxidation. This minimal impact on BDFE(O–H)_avg_ can be attributed to nearly complete thermodynamic compensation resulting from the inverse relationship of cluster nucleophilicity and electrochemical potential.

When the POV-alkoxide cluster surface is directly alkylated at bridging oxygen positions, a statistically significant change in BDFE(O–H)_avg_ is observed. Clusters with the same oxidation state distribution feature hydroxide ligands up to 3 kcal mol^–1^ stronger when compared to their unfunctionalized counterparts. Furthermore, reduction of the surface functionalized cluster produces a larger decrease in O–H bond strength [**1-V**_**6**_**O**_**9**_**(OMe)**_**2**_**(OH)**_**2**_^**–2**^, 66.3 kcal mol ^–1^; **1-V**_**6**_**O**_**7**_**(OMe)**_**2**_**(OH)**_**4**_^**–2**^, 62.6 kcal mol^–1^]. The increased dependence of BDFE(O–H)_avg_ on oxidation state can be attributed to an increased charge localization resulting in O–H bonds whose thermodynamic stability is tightly correlated to the extent of reduction of the cluster.

Ongoing efforts in our laboratory aim to leverage the ability to tune BDFE(O–H)_avg_ of these POV- alkoxide clusters to facilitate proton-coupled electron transfer for small molecule activation. Control of the thermodynamic availability of H atoms at the cluster surface can tune the catalytic activity of hydrogenation reactions.

## Experimental and Computational Methods

### General Considerations

Unless otherwise noted, all manipulations were carried out in the absence of water and oxygen using standard Schlenk techniques or in a UniLab MBraun inert atmosphere drybox under a dinitrogen atmosphere. All glassware was oven-dried for a minimum of 4 h and cooled in an evacuated antechamber prior to use in the drybox. Solvents were dried and deoxygenated on a glass contour system (Pure Process Technology, LLC) and stored over 3 Å molecular sieves purchased from Fisher Scientific and activated prior to use. All clusters were generated according to the literature precedent and bases were purchased from Sigma-Aldrich then converted to their conjugate acids. Polyoxovanadate-alkoxide clusters studied in this work have been synthesized according to previously published procedures.

### Electronic Absorption Spectroscopy

Measurements were recorded at room temperature in anhydrous acetonitrile in a sealed 1 cm quartz cuvette with an Agilent Cary 60 UV–vis spectrophotometer. Elemental analyses were performed on a PerkinElmer 2400 Series II Analyzer, at the Elemental Analysis Facility located at the University of Rochester.

### Cyclic Voltammetry

All experiments were performed using a BioLogic SP 150 potentiostat/galvanostat and the EC-Lab software suite. Glassy carbon discs (3 mm, CH Instruments, USA) were used as working electrodes. Working electrodes were polished using a micro cloth pad and 0.05 μM alumina powder. Potentials recorded during CV were measured relative to a nonaqueous Ag/Ag^+^ reference electrode with 1 mM AgNO_3_ and 0.1 M [^n^Bu_4_N][PF_6_] in acetonitrile (BASi) and ultimately referenced against the Fc^+/0^ couple using an internal reference. A platinum wire served as the counter electrode. All experiments were carried out at room temperature inside a nitrogen-filled glovebox (MBraun, USA). All CV measurements were *iR* compensated at 85% with impedance taken at 100 kHz using the ZIR tool included with the EC-Lab software. CV experiments were conducted at 100 mV s^–1^ on solutions of either 2 or 1 mM analyte and 100 mM [^n^Bu_4_N][PF_6_] supporting electrolyte in acetonitrile.

### Open Circuit Potential

Measurements were recorded with a Bio-Logic SP-150 potentiostat/galvanostat and the EC-Lab software suite. All experiments were performed in a three-electrode system cell configuration that consisted of a glassy-carbon (ø = 3.0 mm) as working electrode (CH Instruments, USA), a Pt wire as the counter electrode (CH Instruments, USA), and an Ag/Ag^+^ nonaqueous reference electrode with 0.01 M AgNO_3_ in 0.1 M [^*n*^Bu_4_N][PF_6_] in acetonitrile (BASi, USA). The supporting electrolyte, [^*n*^Bu_4_N][PF_6_] was purchased from Sigma-Aldrich, recrystallized three times using hot ethanol, and stored under dynamic vacuum for a minimum of 2 days prior to use. All electrochemical measurements were performed at room temperature in a nitrogen-filled drybox. CV cells were prepared with 0.50 mM cluster 1, 0.25 mM cluster 2 (cluster 1 and 2 referring to any pair of clusters differing only by two hydroxy ligands), 0.1 M [^*n*^Bu_4_N][PF_6_], and 50 mM buffer in acetonitrile. OCP was allowed to stabilize (5 min to 1 h) before titration of 100 μL of cluster 2 into the CV cell. Automated titrations were carried out by an NE-1000 One Channel Programmable Syringe Pump for 3–10 repetitions. Upon the conclusion of electrochemical experiments, ferrocene was added to the sample as an internal standard and an additional CV was collected.

### Synthesis of [^n^Bu_4_N]_2_[V_6_O_9_(OH)_2_(OMe)_2_(TRIOL^NO2^)_2_] (**1-V**_**6**_**O**_**9**_**(OMe)**_**2**_**(OH**_**2**_**)**^**–2**^)

A 20 mL scintillation vial was charged with [^n^Bu_4_N]_2_[V_6_O_11_(OMe)_2_(TRIOL^NO2^)_2_] (0.115 g, 0.0873 mmol) and 5 mL DCM. Hydrazobenzene (1 equiv, 0.016 g, 0.087 mmol) was added with stirring. The green solution was stirred for 3 h before the solvent was removed under vacuum. The resulting blue residue was washed with 5 mL ether then 10 mL pentane to afford the desired assembly, **1-V**_**6**_**O**_**9**_**(OMe)**_**2**_**(OH**_**2**_**)**^**–2**^, in quantitative yield. (0.115 g, 0.086 mmol, 99%). The cluster was characterized by electronic absorption spectroscopy which features a broad IVCT band starting at 500 nm: UV–vis [ε(M^–1^ cm^–1^)]: 800 nm (490 M^–1^ cm^–1^) (Figure S1). Fourier transform infrared (FTIR) confirmed the retention of the Lindqvist structure with two distinct absorption bands corresponding to ν(V=O_t_) (945 cm^–1^) and ν(O_b_–CR_3_) (1105 cm^–1^) (Figure S2). Elemental analysis: Calcd for C 41.36% H 7.49% N 5.67% Found: C 41.71% H 7.08% N 5.60%

### Synthesis of [^n^Bu_4_N]_2_[V_6_O_7_(OH)_4_(OMe)_2_(TRIOL^NO2^)_2_] (**1-V**_**6**_**O**_**7**_**(OMe)**_**2**_**(OH)**_**4**_^**–2**^)

A 20 mL scintillation vial was charged with [^n^Bu_4_N]_2_[V_6_O_11_(OMe)_2_(TRIOL^NO2^)_2_] (0.088 g, 0.066 mmol) and 5 mL DCM. Hydrazobenzene (2 equiv, 0.024 g, 0.132 mmol) was added with stirring, resulting in a gradual color change from green to gold over 3 h. The solvent was removed under vacuum to yield a light green oil. The residue was washed 5 mL Et_2_O then 10 mL pentane to afford the desired assembly, **1-V**_**6**_**O**_**7**_**(OMe)**_**2**_**(OH)**_**4**_^**–2**^, in quantitative yield (0.087 g, 0.065 mmol, 99%). The cluster was characterized by electronic absorption spectroscopy which is comparatively featureless with broad band starting at 500 nm UV–vis [ε(M^–1^ cm^–1^)]: 900 nm (490 M^–1^ cm^–1^) (Figure S1). FTIR confirmed the retention of the Lindqvist structure with two distinct absorption bands corresponding to ν(V=O_t_) (945 cm^–1^) and ν(O_b_–CR_3_) (1105 cm^–1^) (Figure S2). Elemental analysis: Calcd for C, 36.53%; H, 6.84%; N, 3.96%. Found: C, 36.47%; H, 6.90%; N, 4.21%

### Computational Methods

DFT calculations were performed using the Gaussian 16 program package.^41,42^ The hybrid functional B3LYP^43^ was used together with the LANL2DZ basis set.^44^ Implicit solvation was utilized via the self-consistent reaction field with acetonitrile as solvent, corresponding to the experiments [SCRF = (SMD,acetonitrile)].^45^ The cluster structures with the counterions (tetramethylammonium instead of tetrabutylammonium in experiments to reduce computational cost) are provided in Supporting Information (Figure S26). All structures were fully optimized to standard tolerances (RMS force ≤3 × 10^–4^ Hartree per Bohr) or even with tighter criteria. Vibrational frequencies were computed for the optimized geometries, confirming the minima to be free of imaginary frequencies. The DFT-optimized structures matched those experimentally resolved via XRD with great agreement (Table S4). Thermochemical values were determined at 298.15 K and 1 atm, corresponding to the experiments. Gibbs free energy values are used to compare the cluster relative energetics. Multiple spin states were taken into consideration with multiplicities ranging from singlet to septet and the lowest energy states were considered. Reduction of the clusters by H atom equivalents was accounted on all possible oxygen sites of the clusters at multiple adsorption configurations and the reported results focused on the most preferred adsorption sites, which were found to be the bridging sites. Reduction of the clusters was found to be exergonic through full occupation of the bridge sites, geometrically congruent with experiments^15,16^ and the V–O_B_, V–O_T_, and V–O_C_ bond lengths increase slightly with H reduction in agreement with experiments^16^ (O_B/T/C_ = bridging, terminal, and central oxides, respectively; Table S4). Natural bond orbital^42^ analysis was used to calculate atomic charges.

## Supporting Information Available

Experimental data including spectroscopic analysis of all clusters synthesized in report, OCP analysis, and cyclic voltammetry data corresponding to data points in potential-p*K*_a_ diagram; and computational results of DFT calculated structures, atomic charges, proton, and electron transfer energetics (PDF)
