# Supplementary material for: Impact of Surface Ligand Identity and Density on the Thermodynamics of H Atom Uptake at Polyoxovanadate-Alkoxide Surfaces
Source: Inorg Chem. 2024 Apr 9;63(16):7206–17. doi: 10.1021/acs.inorgchem.3c04435 (PMC11040723; doi:10.1021/acs.inorgchem.3c04435)
Supplement: Supplementary file 1 — ic3c04435_si_001.pdf [file ic3c04435_si_001.pdf]

Electronic Supporting Information

Impact of Surface Ligand Identity and Density on the Thermodynamics of H-atom Uptake at Polyoxovanadate-alkoxide Surfaces

Kathryn R. Proe[1], Andreas Towarnicky[2], Alex Fertig[1], Zhou Lu[1], Giannis Mpourmpakis[2], and Ellen M. Matson*[1]*

[1]Department of Chemistry, University of Rochester, Rochester NY 14627 USA
*Corresponding author e-mail: matson@chem.rochester.edu

[2]Department of Chemical Engineering, University of Pittsburgh, Pittsburgh, PA 15261, USA
*Corresponding author e-mail: gmpourmp@pitt.edu

Supporting Information Table of Contents

Figure S1. Electronic absorption spectra of $[^nBu_4N]_2[V_6O_9(OH)_2(OMe)_2(TRIOL^{NO2})_2]$ and $[^nBu_4N]_2[V_6O_7(OH)_4(OMe)_2(TRIOL^{NO2})_2]$. S2

Figure S2. FTIR $[^nBu_4N]_2[V_6O_9(OH)_2(OMe)_2(TRIOL^{NO2})_2]$ and of $[^nBu_4N]_2[V_6O_7(OH)_4(OMe)_2(TRIOL^{NO2})_2]$ S2

Figure S3. ESI-MS of $[^nBu_4N]_2[V_6O_9(OH)_2(OMe)_2(TRIOL^{NO2})_2]$ S3

Figure S4. ESI-MS of $[^nBu_4N]_2[V_6O_7(OH)_4(OMe)_2(TRIOL^{NO2})_2]$ S3

Figure S5. OCP plots for **1-$V_6O_{11}(OH)_2$ $^{-2}$** S4

Figure S6. OCP plots **1-$V_6O_9(OH)_4$ $^{-2}$** S4

Figure S7. OCP plots for **1-$V_6O_7(OH)_6$ $^{-2}$** S5

Figure S8. OCP plots for **2-$V_6O_{11}(OH)_2$ $^{-2}$** S5

Figure S9. OCP plots **2-$V_6O_9(OH)_4$ $^{-2}$** S6

Figure S10. OCP plots for **2-$V_6O_7(OH)_6$ $^{-2}$** S6

Figure S11. OCP plots **1-$V_6O_9(OMe)_2(OH)_2$ $^{-2}$** S7

Figure S12. OCP plots **1-$V_6O_7(OMe)_2(OH)_4$ $^{-2}$** S7

Figure S13 CV of $[^nBu_4N]_2[V_6O_9(OH)_4(TRIOL^{NO2})_2]$ S8

Figure S14. CV of $[^nBu_4N]_2[V_6O_{13}(TRIOL^{NO2})_2]$ and $[^nBu_4N]_2[V_6O_{13}(TRIOL^{Me})_2]$ S8

Figures S15-S24. CVs of $[^nBu_4N]_2[V_6O_{13}(TRIOL^{NO2})_2]$ in acetonitrile in the presence of 2 equiv of various organic acids (pKas 5.98 - 39.5) .... S9 - S13

Table S1. List of organic acids used in CV experiments. S14

Figure S25. A plot of experimental BDFE(O-H) $_{avg}$ against the number of V^{IV} centers S14

Figure S26. DFT geometrically optimized structures of $[NMe_4]_2[V_6O_{13}(TRIOL^{NO2})_2]$, $[NMe_4]_2[V_6O_{13}(TRIOL^{Me})_2]$, and $[NMe_4]_2[V_6O_{11}(OMe)_2(TRIOL^{NO2})_2]$. S15

Table S2. DFT calculated BDFE(O-H)$_{avg}$.. .... S16

Table S3. DFT calculated BDFE(O-H) values.. .... S16

Table S4. Experimentally measured[1] and DFT-calculated bond distances, for **1-$V_6O_{13-x}(OH)_x$ $^{-2}$** S17

Figure S27. DFT-calculated electron and proton transfer free energies. S18

Figure S28. Atomic charge trends with H reduction. S19

Figure S29. Atom labels used in NBO tables. S19

Table S5. NBO atomic charges for **1-$V_6O_{13-x}(OH)_x$ $^{-2}$** as a function of H reduction S20

Table S6. NBO atomic charges for **2-$V_6O_{13-x}(OH)_x$ $^{-2}$** as a function of H reduction S21

Table S7. NBO atomic charges for **1-$V_6O_{13-x}(OMe)_2(OH)_x$ $^{-2}$** as a function of H reduction S22

Figure S30. OCP experimental time traces S23

References. S24

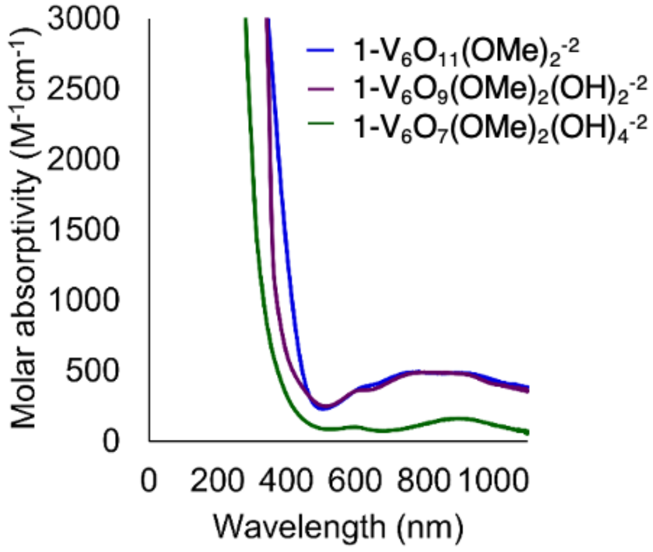

Figure S1. Electronic absorption spectra of $[^nBu_4N]_2[V_6O_{11}(OMe)_2(TRIOL^{NO2})_2]$ (blue), $[^nBu_4N]_2[V_6O_9(OH)_2(OMe)_2(TRIOL^{NO2})_2]$ (purple), and of $[^nBu_4N]_2[V_6O_7(OH)_4(OMe)_2(TRIOL^{NO2})_2]$ (green) in acetonitrile collected at room temperature (21 ^oC).

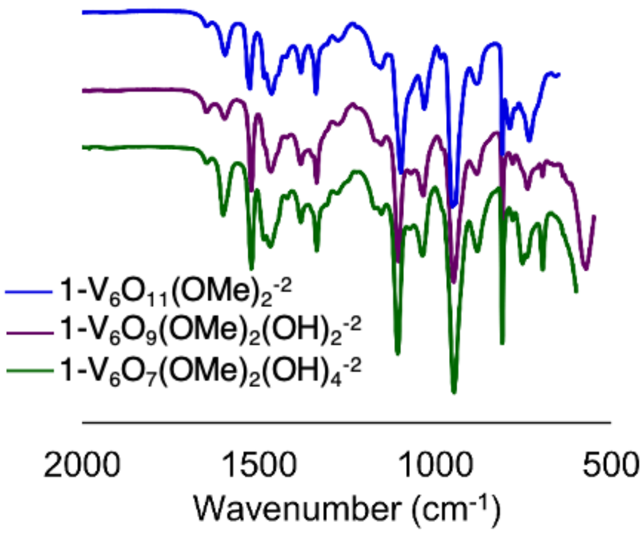

Figure S2. Infrared Spectra of $[^nBu_4N]_2[V_6O_{11}(OMe)_2(TRIOL^{NO2})_2]$ (blue), $[^nBu_4N]_2[V_6O_9(OH)_2(OMe)_2(TRIOL^{NO2})_2]$ (purple), and $[^nBu_4N]_2[V_6O_7(OH)_4(OMe)_2(TRIOL^{NO2})_2]$ (green)

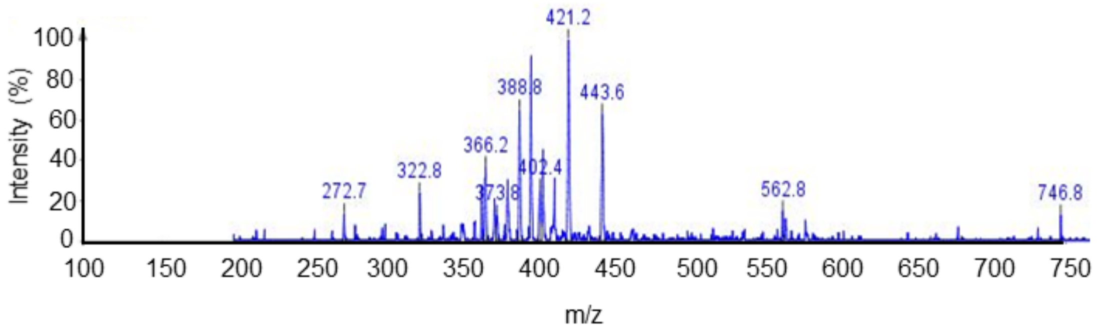

Figure S3. ESI-MS of [$^{n}Bu_4N]_2[V_6O_9(OH)_2(OMe)_2(TRIOL^{NO2})_2$] (**1-$V_6O_9(OMe)_2(OH)_2^{-2}$**).

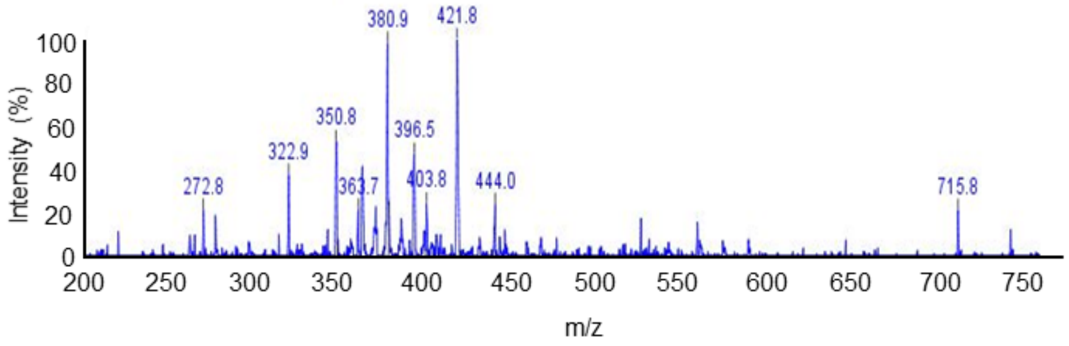

Figure S4. ESI-MS of [$^{n}Bu_4N]_2[V_6O_7(OH)_4(OMe)_2(TRIOL^{NO2})_2$] (**1-$V_6O_7(OMe)_2(OH)_4^{-2}$**).

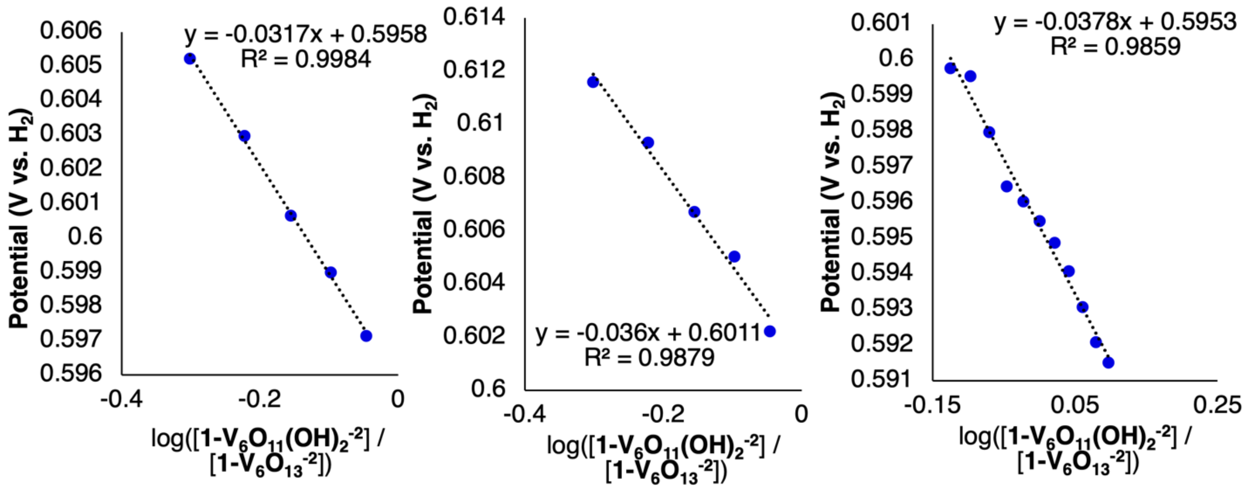

Figure S5. Plots of the OCP values referenced against H_2 measured at various ratios of **1-$V_6O_{11}(OH)_2^{-2}$**:**1-$V_6O_{13}^{-2}$** against the log of the ratio of the concentrations of clusters (three trials). All measurements were performed in acetonitrile containing a 0.05 M buffer of 1:1 DMA/DMAH$^+$ (pKa(DMAH$^+$) = 11.47) and supporting electrolyte (0.1 M [^{n}Bu$_4$N][PF$_6$]). The slope of the line closely resembles the value expected by the Nernst equation for a 2H$^+$/2e$^-$ process. From the y-intercept (see equation), the BDFE(O-H)$_{avg}$ describing the loss of the first two H-atom equivalents from the surface of **1-$V_6O_{11}(OH)_2^{-2}$** is calculated using Eqn 2 (main text).

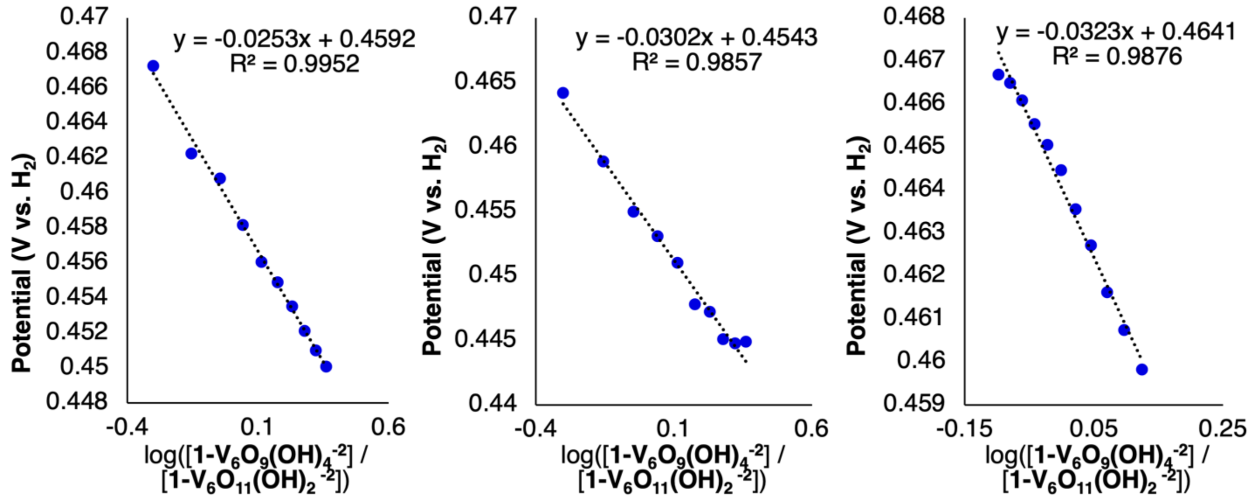

Figure S6. Plots of the OCP values referenced against H_2 measured at various ratios of **1-$V_6O_9(OH)_4^{-2}$**:**1-$V_6O_{11}(OH)_2^{-2}$** against the log of the ratio of the concentrations of clusters (three trials). All measurements were performed in acetonitrile containing a 0.05 M buffer of 1:1 TEA/TEAH$^+$ (pKa(TEAH$^+$) = 18.83) and supporting electrolyte (0.1 M [^{n}Bu$_4$N][PF$_6$]). The slope of the line closely resembles the value expected by the Nernst equation for a 2H$^+$/2e$^-$ process. From the y-intercept (see equation), the BDFE(O-H)$_{avg}$ describing the loss of the first two H-atom equivalents from the surface of **1-$V_6O_9(OH)_4^{-2}$** is calculated using Eqn 2 (main text).

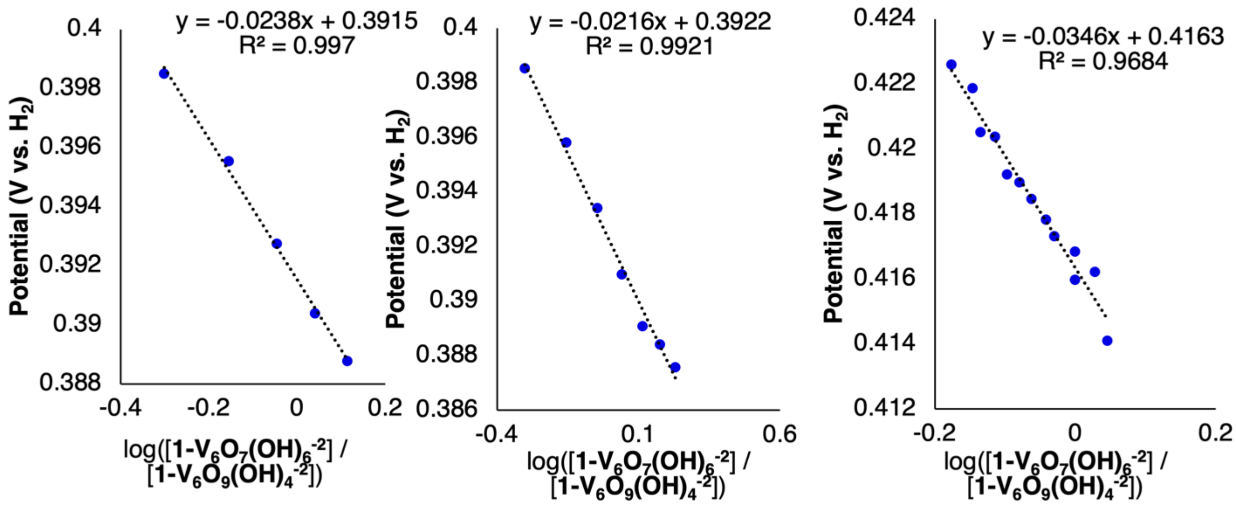

Figure S7. Plots of the OCP values referenced against H_2 measured at various ratios of **1-$V_6O_7(OH)_6^{-2}$**:**1-$V_6O_9(OH)_4^{-2}$** against the log of the ratio of the concentrations of clusters (three trials). All measurements were performed in acetonitrile containing a 0.05 M buffer of 1:1 TMG/$TMGH^+$ (pKa($TMGH^+$) = 23.35) and supporting electrolyte (0.1 M [$^n Bu_4N$][PF_6]). The slope of the line closely resembles the value expected by the Nernst equation for a $2H^+/2e^-$ process. From the y-intercept (see equation), the BDFE(O-H)$_{avg}$ describing the loss of the first two H-atom equivalents from the surface of **1-$V_6O_7(OH)_6^{-2}$** is calculated using Eqn 2 (main text).

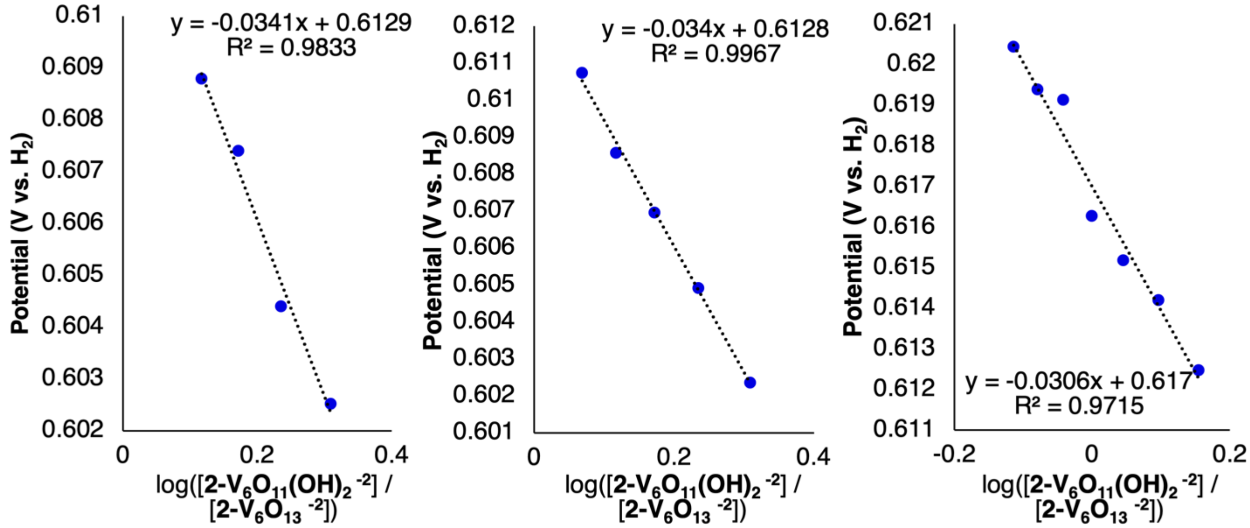

Figure S8. Plots of the OCP values referenced against H_2 measured at various ratios of **2-$V_6O_{11}(OH)_2^{-2}$**:**2-$V_6O_{13}^{-2}$** against the log of the ratio of the concentrations of clusters (three trials). All measurements were performed in acetonitrile containing a 0.05 M buffer of 1:1 Pyrd/$PyrdH^+$ (pKa($PyrdH^+$) = 12.53) and supporting electrolyte (0.1 M [$^n Bu_4N$][PF_6]). The slope of the line closely resembles the value expected by the Nernst equation for a $2H^+/2e^-$ process. From the y-intercept (see equation), the BDFE(O-H)$_{avg}$ describing the loss of the first two H-atom equivalents from the surface of **2-$V_6O_{11}(OH)_2^{-2}$** is calculated using Eqn 2 (main text).

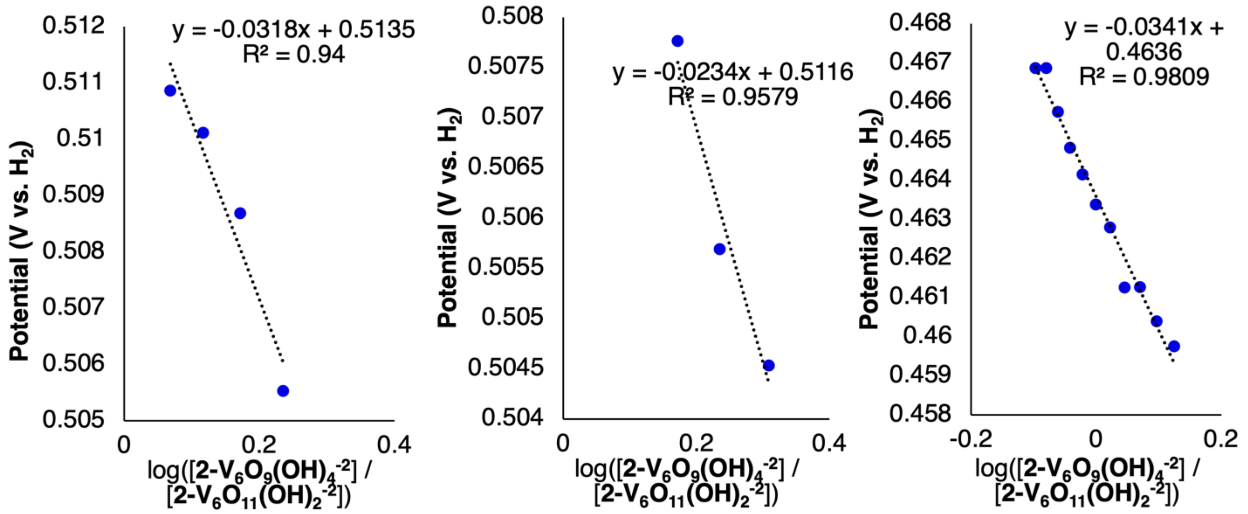

Figure S9. Plots of the OCP values referenced against H_2 measured at various ratios of **2-$V_6O_9(OH)_4^{-2}$**:**2-$V_6O_{11}(OH)_2^{-2}$** against the log of the ratio of the concentrations of clusters (three trials). All measurements were performed in acetonitrile containing a 0.05 M buffer of 1:1 TEA/$TEAH^+$ (pKa($TEAH^+$) = 18.83) and supporting electrolyte (0.1 M [$^nBu_4N$][PF_6]). The slope of the line closely resembles the value expected by the Nernst equation for a $2H^+/2e^-$ process. From the y-intercept (see equation), the BDFE(O-H)$_{avg}$ describing the loss of the first two H-atom equivalents from the surface of **2-$V_6O_9(OH)_4^{-2}$** is calculated using Eqn 2 (main text).

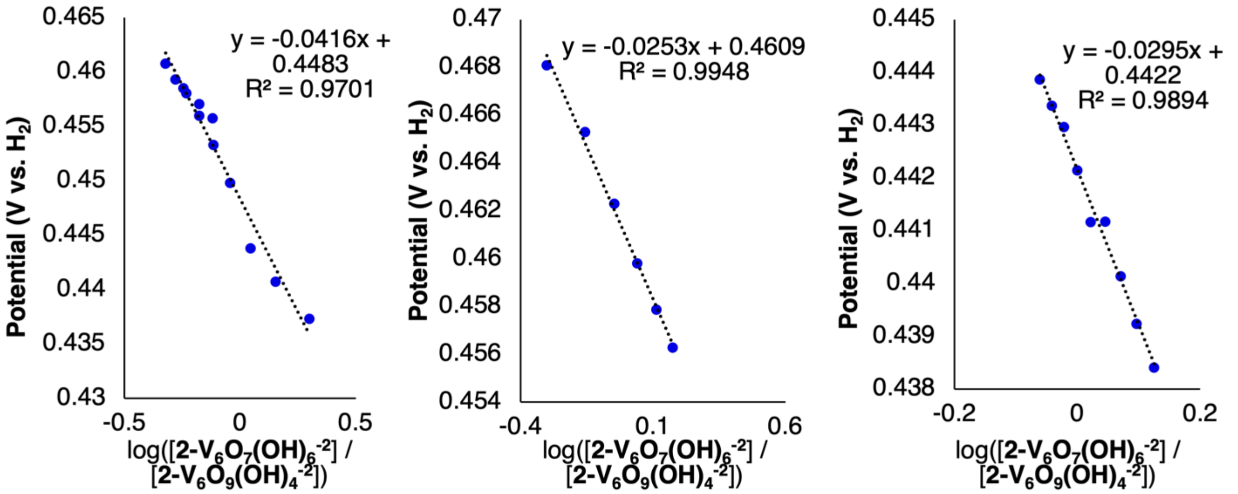

Figure S10. Plots of the OCP values referenced against H_2 measured at various ratios of **2-$V_6O_7(OH)_6^{-2}$**:**2-$V_6O_9(OH)_4^{-2}$** against the log of the ratio of the concentrations of clusters (three trials). All measurements were performed in acetonitrile containing a 0.05 M buffer of 1:1 TMG/$TMGH^+$ (pKa($TMGH^+$) = 23.35) and supporting electrolyte (0.1 M [nBu_4N][PF_6]). The slope of the line closely resembles the value expected by the Nernst equation for a $2H^+/2e^-$ process. From the y-intercept (see equation), the BDFE(O-H)$_{avg}$ describing the loss of the first two H-atom equivalents from the surface of **2-$V_6O_7(OH)_6^{-2}$** is calculated using Eqn 2 (main text).

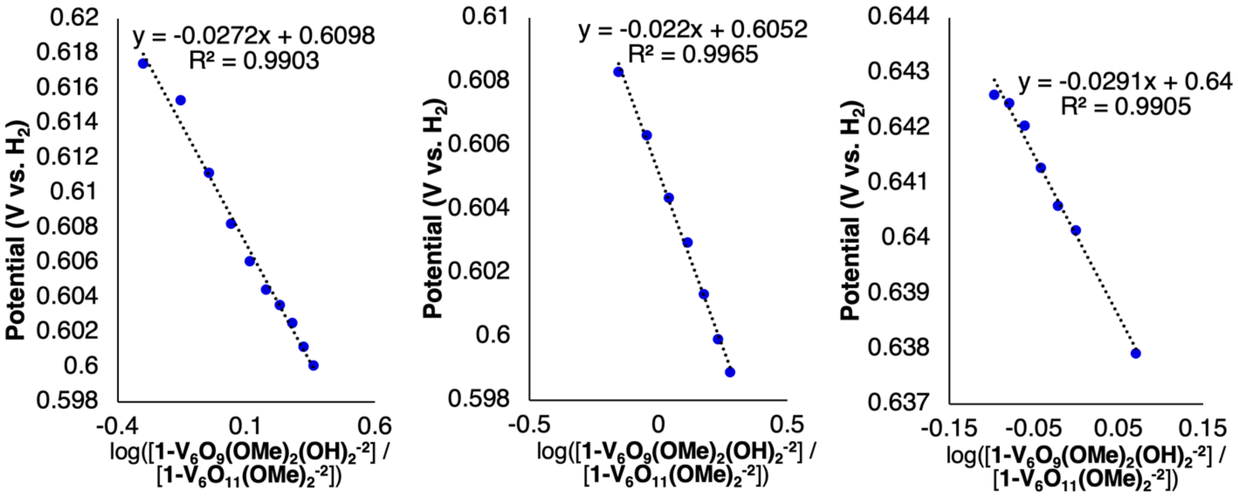

Figure S11. Plots of the OCP values referenced against H_2 measured at various ratios of **1-$V_6O_9(OMe)_2(OH)_2^{-2}$**:**1-$V_6O_{11}(OMe)_2^{-2}$** against the log of the ratio of the concentrations of clusters (three trials). All measurements were performed in acetonitrile containing a 0.05 M buffer of 1:1 ClPyrd/ClPyrdH$^+$ (pKa(ClPyrdH$^+$) = 6.79) and supporting electrolyte (0.1 M [^{n}Bu$_4$N][PF$_6$]). The slope of the line closely resembles the value expected by the Nernst equation for a 2H$^+$/2e$^-$ process. From the y-intercept (see equation), the BDFE(O-H)$_{avg}$ describing the loss of the first two H-atom equivalents from the surface of **1-$V_6O_9(OMe)_2(OH)_2^{-2}$** is calculated using Eqn 2 (main text).

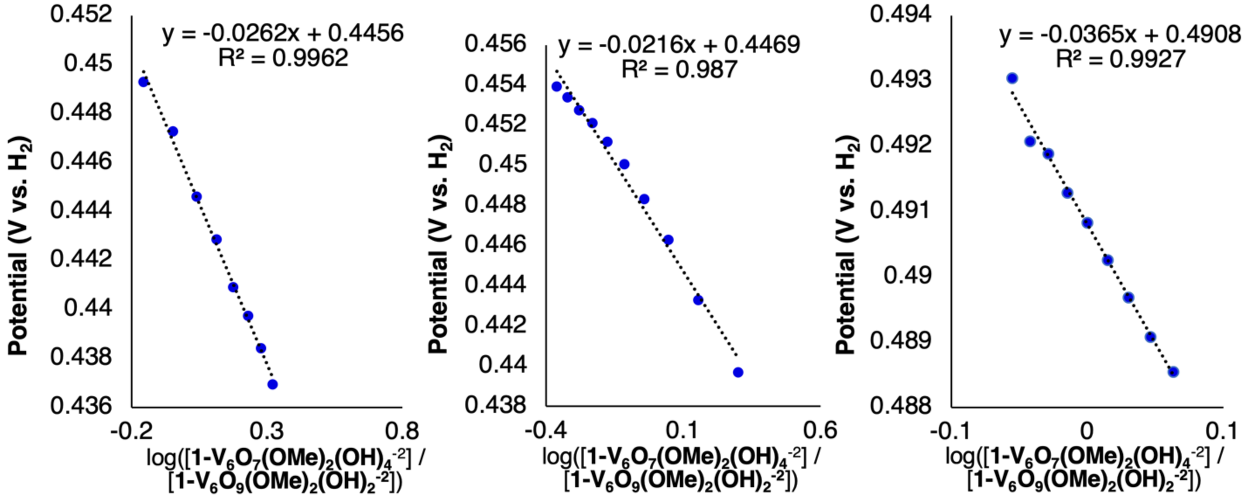

Figure S12. Plots of the OCP values referenced against H_2 measured at various ratios of **1-$V_6O_7(OMe)_2(OH)_4$ $^{-2}$/$V_6O_9(OMe)_2(OH)_2^{-2}$** against the log of the ratio of the concentrations of clusters (three trials). All measurements were performed in acetonitrile containing a 0.05 M buffer of 1:1 Pyrd/PyrdH$^+$ (pKa(PyrdH$^+$) = 12.53) and supporting electrolyte (0.1 M [^{n}Bu$_4$N][PF$_6$]). The slope of the line closely resembles the value expected by the Nernst equation for a 2H$^+$/2e$^-$ process. From the y-intercept (see equation), the BDFE(O-H)$_{avg}$ describing the loss of the first two H-atom equivalents from the surface of **1-$V_6O_7(OMe)_2(OH)_4$ $^{-2}$** is calculated using Eqn 2 (main text).

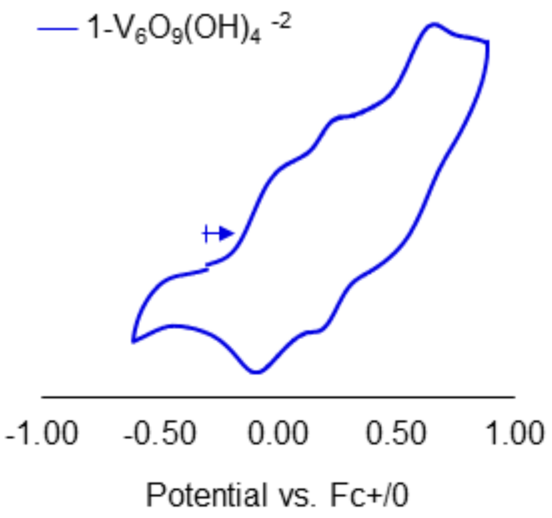

Figure S13. Cyclic voltammogram of [^{n}Bu$_4$N]$_2$[$V_6O_9(OH)_4$(TRIOL^{NO2})$_2$] collected using 1 mM solution of the cluster in acetonitrile with 0.1 M [^{n}Bu$_4$N][PF_6] as the supporting electrolyte at a scan rate of 100 mV s^{-1}.

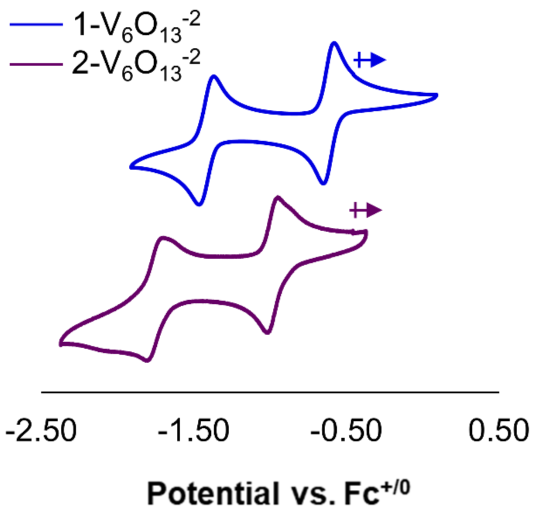

Figure S14. Cyclic voltammogram of [^{n}Bu$_4$N]$_2$[V_6O_{13}(TRIOL^{NO2})$_2$] (blue) and [^{n}Bu$_4$N]$_2$[V_6O_{13}(TRIOL^{Me})$_2$] (purple) collected using 1 mM solution of the cluster in acetonitrile with 0.1 M [nBu$_4$N][PF_6] as the supporting electrolyte at a scan rate of 100 mV s^{-1}. The change in peripheral ligands results in a shift of nearly 0.3V vs Fc$^{+/0}$.

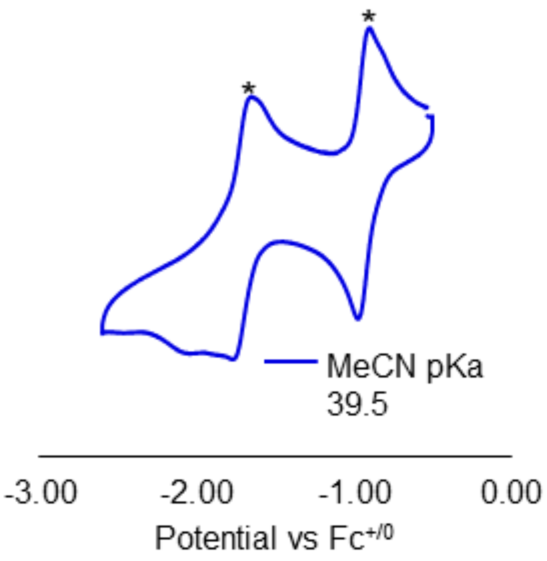

Figure S15. Cyclic voltammogram of $[^nBu_4N]_2[V_6O_{13}(TRIOL^{Me})_2]$ collected using 1 mM solution of the cluster in acetonitrile (pKa 39.5) with 0.1 M $[^nBu_4N][PF_6]$ as the supporting electrolyte at a scan rate of 100 mV s^{-1}.

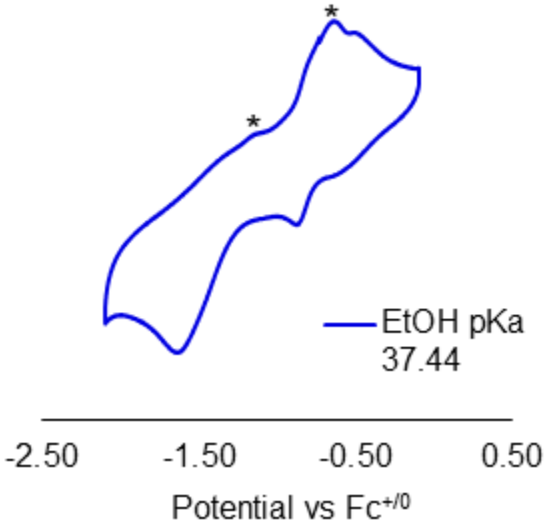

Figure S16. Cyclic voltammogram of $[^nBu_4N]_2[V_6O_{13}(TRIOL^{Me})_2]$ collected using 1 mM solution of the cluster and 2mM EtOH (pKa 37.44) in acetonitrile with 0.1 M $[^nBu_4N][PF_6]$ as the supporting electrolyte at a scan rate of 100 mV s^{-1}.

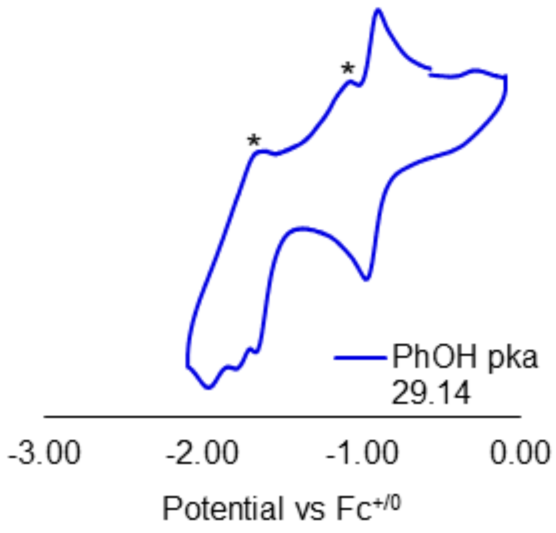

Figure S17. Cyclic voltammogram of $[^nBu_4N]_2[V_6O_{13}(TRIOL^{Me})_2]$ collected using 1 mM solution of the cluster and 2mM PhOH (pKa 29.14) in acetonitrile with 0.1 M $[^nBu_4N][PF_6]$ as the supporting electrolyte at a scan rate of 100 mV s^{-1}.

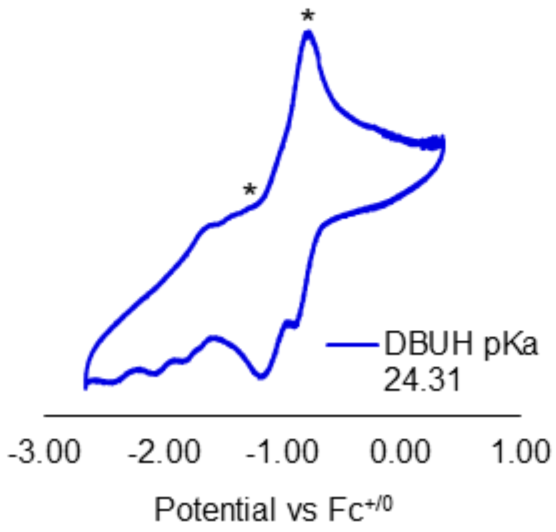

Figure S18. Cyclic voltammogram of $[^nBu_4N]_2[V_6O_{13}(TRIOL^{Me})_2]$ collected using 1 mM solution of the cluster and 2mM $DBUH^+$ (pKa 24.31) in acetonitrile with 0.1 M $[^nBu_4N][PF_6]$ as the supporting electrolyte at a scan rate of 100 mV s^{-1}.

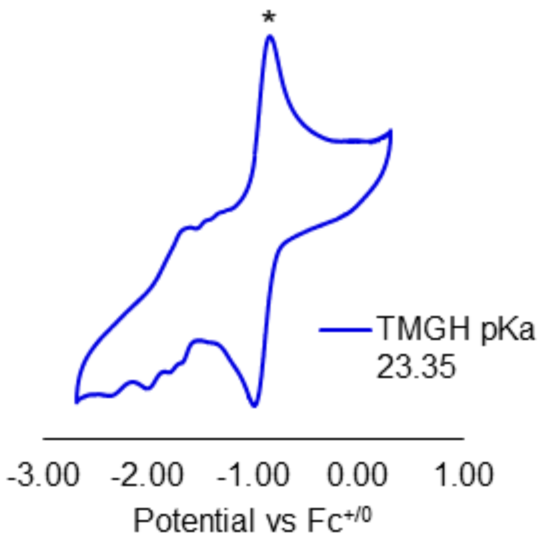

Figure S19. Cyclic voltammogram of $[^nBu_4N]_2[V_6O_{13}(TRIOL^{Me})_2]$ collected using 1 mM solution of the cluster and 2mM $TMGH^+$ (pKa 23.35) in acetonitrile with 0.1 M $[^nBu_4N][PF_6]$ as the supporting electrolyte at a scan rate of 100 mV s^{-1}.

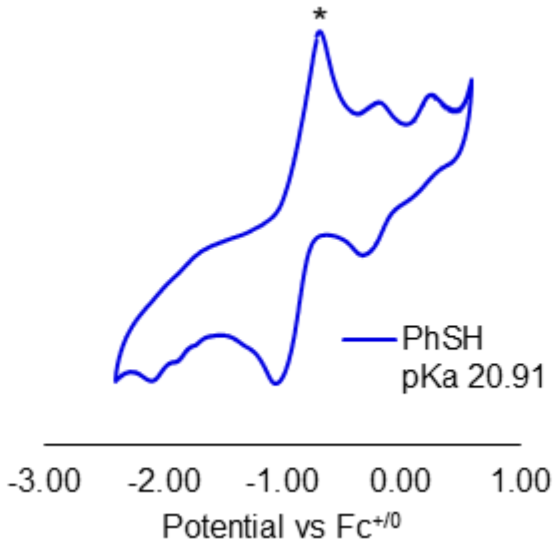

Figure S20. Cyclic voltammogram of $[^nBu_4N]_2[V_6O_{13}(TRIOL^{Me})_2]$ collected using 1 mM solution of the cluster and 2mM PhSH (pKa 20.91) in acetonitrile with 0.1 M $[^nBu_4N][PF_6]$ as the supporting electrolyte at a scan rate of 100 mV s^{-1}.

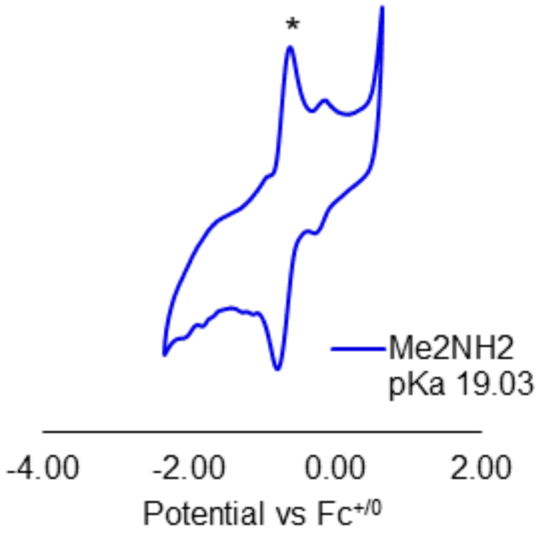

Figure S21. Cyclic voltammogram of $[^nBu_4N]_2[V_6O_{13}(TRIOL^{Me})_2]$ collected using 1 mM solution of the cluster and 2mM $Me_2NH_2^+$ (pKa 19.03) in acetonitrile with 0.1 M $[^nBu_4N][PF_6]$ as the supporting electrolyte at a scan rate of 100 mV s^{-1}.

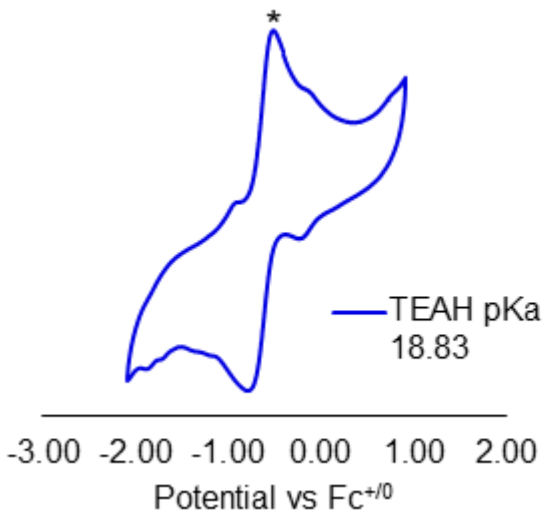

Figure S22. Cyclic voltammogram of $[^nBu_4N]_2[V_6O_{13}(TRIOL^{Me})_2]$ collected using 1 mM solution of the cluster and 2mM $TEAH^+$ (pKa 18.83) in acetonitrile with 0.1 M $[^nBu_4N][PF_6]$ as the supporting electrolyte at a scan rate of 100 mV s^{-1}.

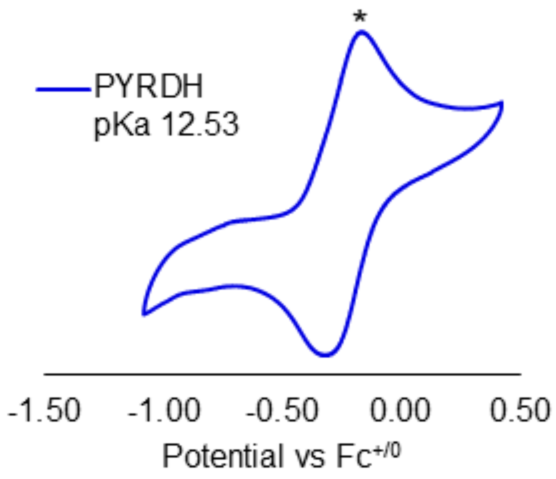

Figure S23. Cyclic voltammogram of $[^nBu_4N]_2[V_6O_{13}(TRIOL^{Me})_2]$ collected using 1 mM solution of the cluster and 2mM $PyrdH^+$ (pKa 12.53) in acetonitrile with 0.1 M $[^nBu_4N][PF_6]$ as the supporting electrolyte at a scan rate of 100 mV s^{-1}.

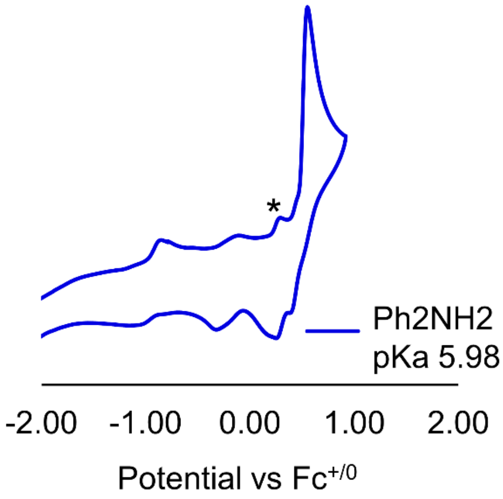

Figure S24. Cyclic voltammogram of $[^nBu_4N]_2[V_6O_{13}(TRIOL^{Me})_2]$ collected using 1 mM solution of the cluster and 2mM $Ph_2NH_2^+$ (pKa 5.98) in acetonitrile with 0.1 M $[^nBu_4N][PF_6]$ as the supporting electrolyte at a scan rate of 100 mV s^{-1}.

Table S1. List of organic acids used in cyclic voltammetry experiments, along with the acid dissociation constants used for this study. [1-6]

Acid	Abbreviation	pK_a	pK_a Reference
Diphenylammonium tetrafluoroborate	$Ph_2NH_2^+$	5.98	1,2
2-Chloropyridinium tetrafluoroborate	2-ClPyrdH$^+$	6.79	3
N,N-Dimethylanilinium tetrafluoroborate	N,N-DMAH$^+$	11.47	3
Pyridinium tetrafluoroborate	PyrdH$^+$	12.53	3
Triethylammonium tetrafluoroborate	TEAH$^+$	18.83	3
Dimethylammonium chloride	$Me_2NH_2^+$	19.03	3
Thiophenol	PhSH	20.91	4,1
1,1,3,3-Tetramethylguanidinium tetrafluoroborate	TMGH$^+$	23.35	3
DBU	DBUH$^+$	24.31	3
Phenol	PhOH	29.14	5
Ethanol	EtOH	37.44	1,4
Acetonitrile	MeCN	39.5	1,6

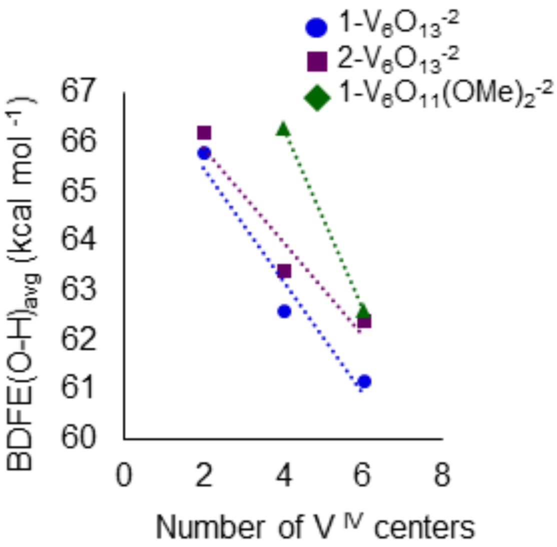

Figure S25. A plot of experimental BDFE(O-H) $_{avg}$ against the number of V^{IV} centers in the reduced clusters demonstrates the correlation between cluster oxidation state and the strength of O-H bonds at the surface with more reduced clusters having more reactive H-atoms.

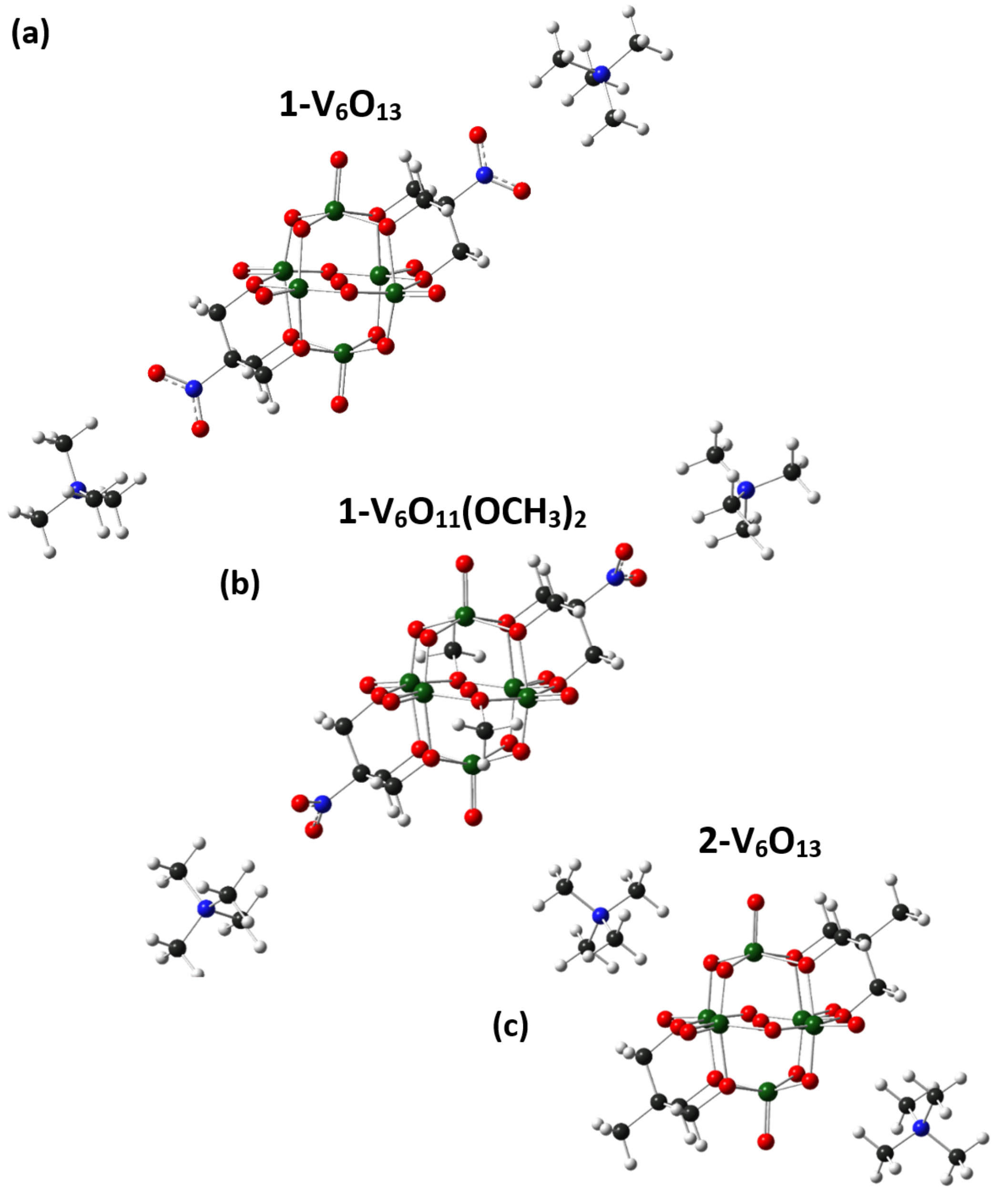

Figure S26. DFT geometrically optimized structures of: (a) $[NMe_4]_2[V_6O_{13}(TRIOL^{NO2})_2]$ (**$1\text{-}V_6O_{13}^{-2}$**), (b) $[NMe_4]_2[V_6O_{11}(OMe)_2(TRIOL^{NO2})_2]$ (**$1\text{-}V_6O_{11}(OCH_3)_2^{-2}$**), (c) $[NMe_4]_2[V_6O_{13}(TRIOL^{Me})_2]$ (**$2\text{-}V_6O_{13}^{-2}$**). Key: dark green spheres, V; red spheres, O; black spheres, C; blue spheres, N; white-gray spheres, H.

Table S2. *DFT calculated BDFE(O-H)$_{avg}$.*

Cluster	DFT BDFE(O-H)$_{avg}$ kcal mol^{-1}
**1-$V_6O_{11}(OH)_2^{-2}$**	67.2
**1-$V_6O_9(OH)_4^{-2}$**	66.2
**1-$V_6O_7(OH)_6^{-2}$**	65.6
**2-$V_6O_{11}(OH)_2^{-2}$**	65.9
**2-$V_6O_9(OH)_4^{-2}$**	64.6
**2-$V_6O_7(OH)_6^{-2}$**	64.2
**1-$V_6O_9(OMe)_2(OH)_2^{-2}$**	67.0
**1-$V_6O_7(OMe)_2(OH)_4^{-2}$**	64.7

Table S3. *DFT calculated BDFE(O-H).* Values calculated per Eqn. S1, below:

$$BDFE(\,O-H)_x^{Cluster} = G_{(x-1)H}^{Cluster} + G(H^\bullet) \,-\, G_{xH}^{Cluster} \qquad \text{Eqn. S1}$$

Cluster	DFT BDFE(O-H) kcal mol^{-1}
**1-$V_6O_{12}(OH)_1^{-2}$**	66.3
**1-$V_6O_{11}(OH)_2^{-2}$**	68.1
**1-$V_6O_{10}(OH)_3^{-2}$**	66.1
**1-$V_6O_9(OH)_4^{-2}$**	66.2
**1-$V_6O_8(OH)_5^{-2}$**	65.6
**1-$V_6O_7(OH)_6^{-2}$**	65.7
**2-$V_6O_{12}(OH)_1^{-2}$**	64.7
**2-$V_6O_{11}(OH)_2^{-2}$**	67.1
**2-$V_6O_{10}(OH)_3^{-2}$**	65.2
**2-$V_6O_9(OH)_4^{-2}$**	64.1
**2-$V_6O_8(OH)_5^{-2}$**	64.6
**2-$V_6O_7(OH)_6^{-2}$**	63.8
**1-$V_6O_{10}(OMe)_2(OH)_1^{-2}$**	67.5
**1-$V_6O_9(OMe)_2(OH)_2^{-2}$**	66.5
**1-$V_6O_8(OMe)_2(OH)_3^{-2}$**	65.6
**1-$V_6O_7(OMe)_2(OH)_4^{-2}$**	63.8

Table S4. Experimentally measured[7] and DFT-calculated bond distances, for **1-$V_6O_{13-x}(OH)_x^{-2}$**. Distances are between Vanadiums and the respective adjacent atoms. O_C = Oxygen at Center of cluster. O_b = Bridge Oxygen. $(OH)_b$ = Bridge OH. O_t = Terminal Oxygen. O_L = TRIOL Ligand Oxygen.

**1-$V_6O_{13-x}(OH)_x^{-2}$**	**Experimental Measurements**			**DFT Computational Results**		
	**$(OH)_0$**	**$(OH)_4$**	**$(OH)_6$**	**$(OH)_0$**	**$(OH)_4$**	**$(OH)_6$**
Bond:	Bond Distance, Angstroms			Bond Distance, Angstroms		
V–O_C	2.2460	2.2899	2.3038	2.2659	2.3139	2.3328
V–O_b	1.8175	1.8855	–	1.8079	1.9077	–
V–$(OH)_b$	–	1.9780	1.9942	--	1.9474	1.9900
V=O_t	1.6030	1.6117	1.6106	1.6057	1.6139	1.6169
V–O_L	2.0275	2.0161	2.0135	2.0293	2.0246	2.0179

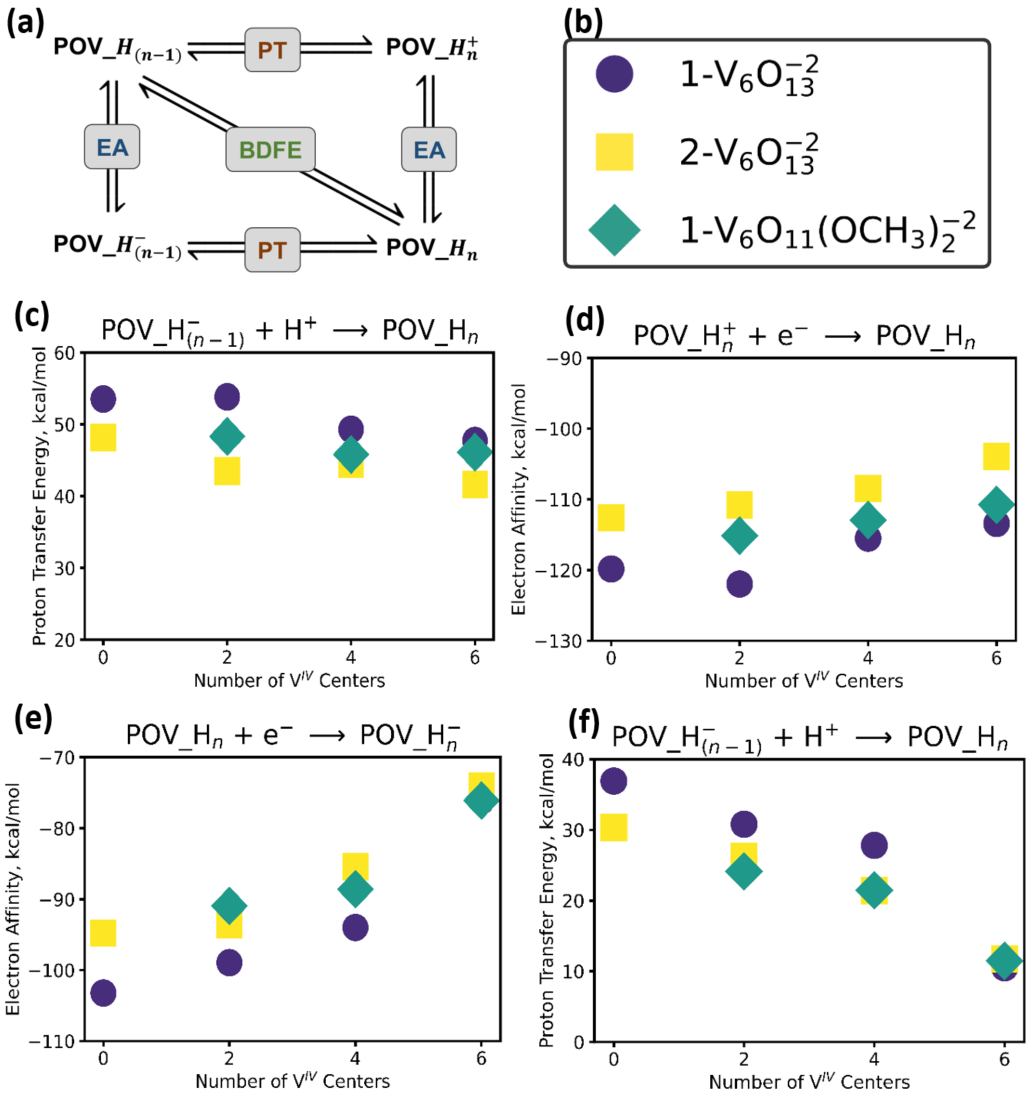

Figure S27. DFT-calculated electron and proton transfer free energies. In this thermodynamic cycle, the reference for the proton transfer is to the hydrogen atom; thus the proton transfer reaction free energies include an adjustment term of – [G(H$^{\bullet}$) – G(H^{+})]. The sum of the electron and proton transfer energies comprise the respective BDFE(O-H)$_{avg}$ for 2H/4H/6H (n=2,4,6). For the 0H clusters, the electron and proton transfer energies are calculated from the 0H state (n=1). The DFT charge transfer values are calculated from the specific n = 2, 4, 6 clusters (e.g; not from the average of values for x = 1 & x = 2). Blue circles: $[V_6O_{13-x}(OH)_x(TRIOL^{NO2})_2]^{-2}$. Yellow squares: $[V_6O_{13-x}(OH)_x(TRIOL^{CH3})_2]^{-2}$. Green diamonds: $[V_6O_{11-x}(OCH_3)_2(OH)_x(TRIOL^{NO2})_2]^{-2}$. (a) Square Scheme[8, 9] , PT = Proton Transfer Energy, EA = Electron Affinity (representing electron transfer), (b) color and symbol for each different cluster series, (c) proton transfer energies corresponding to the top of the square scheme, (d) electron affinities corresponding to the right side of the square scheme, (e) electron affinities corresponding to the left of the square scheme, (f) proton transfer energies corresponding to the bottom of the square scheme.

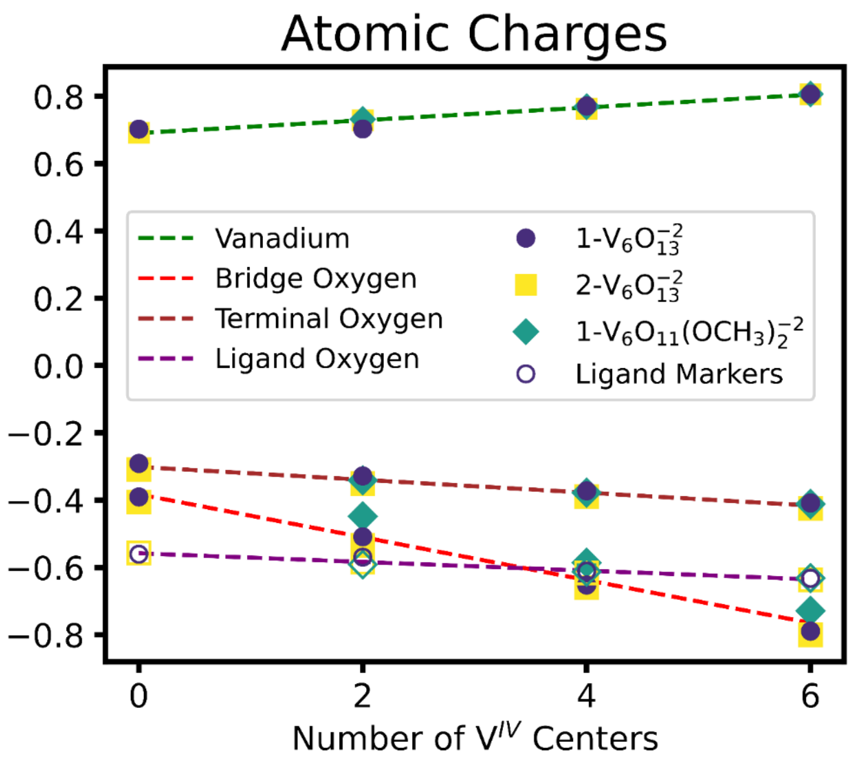

Figure S28. Atomic charge trends with H reduction. Detailed results are presented in **Tables S5-7**. Charge from H reduction is partially localized on the corresponding bridge oxygen(s).

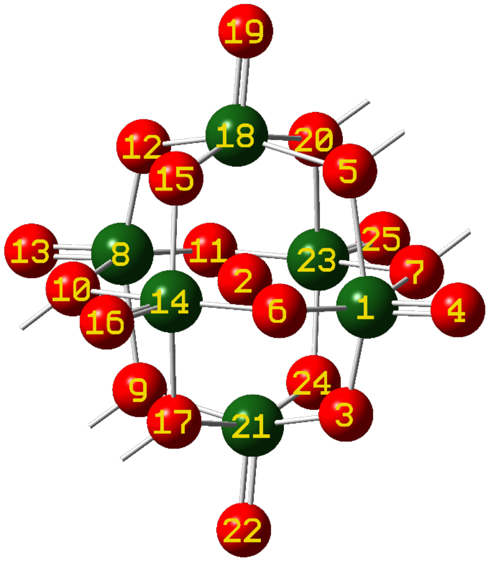

Figure S29. Atom labels used in the **Tables S5-7** for atomic charges. These labels are the same for all different clusters.

Table S5. NBO atomic charges for **1-$V_6O_{13-x}(OH)_x^{-2}$** as a function of H reduction. The atom numbers are shown in Figure S29.

**NBO Atomic Charges of 1-$V_6O_{13-x}(OH)_x^{-2}$**				
**No. of V^{IV} centers**	**0**	**2**	**4**	**6**
**Vanadium**				
V1	0.70300	0.78566	0.78668	0.81307
V8	0.70300	0.73219	0.79688	0.78990
V14	0.70331	0.71771	0.72663	0.80340
V18	0.70325	0.77084	0.77258	0.80947
V21	0.70326	0.70083	0.73153	0.80309
V23	0.70331	0.71132	0.81204	0.80130
$V_{average}$	0.70319	0.73643	0.77106	0.80337
**Bridge Oxygens**				
O3	-0.38969	-0.41779	-0.41412	-0.72964
O6	-0.38987	-0.73773	-0.73775	-0.74418
O11	-0.38987	-0.38408	-0.78675	-0.77650
O12	-0.38969	-0.72866	-0.79629	-0.75541
O15	-0.38974	-0.40701	-0.40558	-0.75433
O24	-0.38974	-0.39837	-0.73968	-0.76334
Bridge $O_{average}$	-0.38977	-0.51227	-0.64670	-0.75390
**Terminal Oxygens**				
O4	-0.28868	-0.37737	-0.38954	-0.32508
O13	-0.28868	-0.29849	-0.39615	-0.36649
O16	-0.28911	-0.32251	-0.33075	-0.33840
O19	-0.28937	-0.38229	-0.39010	-0.32950
O22	-0.28937	-0.32726	-0.33190	-0.35686
O25	-0.28911	-0.30076	-0.39379	-0.36808
Terminal $O_{average}$	-0.28905	-0.33478	-0.37204	-0.34740
**TRIOL Ligand Bonded Oxygens**				
O5	-0.56081	-0.61716	-0.62059	-0.54211
O7	-0.56042	-0.58032	-0.62283	-0.62799
O9	-0.56081	-0.55939	-0.63138	-0.58389
O10	-0.56042	-0.58156	-0.58496	-0.60646
O17	-0.56059	-0.59146	-0.59073	-0.5922
O20	-0.56059	-0.60339	-0.62651	-0.61243
Ligand $O_{average}$	-0.56061	-0.58888	-0.61283	-0.59418

Table S6. NBO atomic charges for **2-$V_6O_{13-x}(OH)_x^{-2}$** as a function of H reduction. The atom numbers are shown in Figure S29.

NBO Atomic Charges of 2-$V_6O_{13-x}(OH)_x^{-2}$				
**No. of V^{IV} centers**	**0**	**2**	**4**	**6**
**Vanadium**				
V1	0.69208	0.69073	0.71982	0.80622
V8	0.69209	0.71641	0.71222	0.80092
V14	0.68990	0.69798	0.80404	0.79618
V18	0.69603	0.70768	0.76628	0.80440
V21	0.69602	0.76242	0.76697	0.81364
V23	0.68990	0.79611	0.80930	0.80953
$V_{average}$	0.69267	0.72856	0.76311	0.80515
**Bridge Oxygens**				
O3	-0.39903	-0.42252	-0.41983	-0.79510
O6	-0.41785	-0.42146	-0.75326	-0.80211
O11	-0.41784	-0.74797	-0.75223	-0.79399
O12	-0.39903	-0.41374	-0.43367	-0.80355
O15	-0.39607	-0.40578	-0.80063	-0.81028
O24	-0.39607	-0.79862	-0.80054	-0.79471
Bridge $O_{average}$	-0.40432	-0.53502	-0.66003	-0.79996
**Terminal Oxygens**				
O4	-0.30562	-0.33886	-0.34544	-0.42367
O13	-0.30562	-0.31458	-0.34218	-0.42840
O16	-0.31935	-0.33499	-0.41640	-0.42989
O19	-0.30895	-0.31344	-0.41219	-0.41747
O22	-0.30894	-0.40211	-0.41222	-0.42186
O25	-0.31935	-0.40245	-0.40820	-0.42765
Terminal $O_{average}$	-0.31131	-0.35107	-0.38944	-0.42482
**TRIOL Ligand Bonded Oxygens**				
O5	-0.55336	-0.55728	-0.6367	-0.63805
O7	-0.55182	-0.5856	-0.58289	-0.63629
O9	-0.55336	-0.62063	-0.63371	-0.63676
O10	-0.55182	-0.54498	-0.5817	-0.63529
O17	-0.56411	-0.58696	-0.63078	-0.64223
O20	-0.56412	-0.60937	-0.63151	-0.63315
Ligand $O_{average}$	-0.55643	-0.58414	-0.61622	-0.63696

Table S7. NBO atomic charges for **1-$V_6O_{11-X}(OMe)_2(OH)_X^{-2}$** as a function of H reduction**.** The atom numbers are shown in Figure S29

**NBO Atomic Charges of 1-$V_6O_{11-X}(OMe)_2(OH)_X^{-2}$**			
**No. of V^{IV} Centers**	**2**	**4**	**6**
**Vanadium**			
V1	0.70332	0.73861	0.81916
V8	0.70332	0.78355	0.81836
V14	0.76677	0.77675	0.80368
V18	0.72721	0.72262	0.80046
V21	0.72721	0.80227	0.79834
V23	0.76677	0.79521	0.80372
$V_{average}$	0.73243	0.76984	0.80729
**Bridge Oxygens**			
O3	-0.39238	-0.73895	-0.78871
O6	-0.41487	-0.41218	-0.78411
O11	-0.41487	-0.78970	-0.78439
O12	-0.39238	-0.41484	-0.78938
O15	-0.53428	-0.54854	-0.61205
O24	-0.53428	-0.61004	-0.61004
Bridge $O_{average}$	-0.44718	-0.58571	-0.72811
**Terminal Oxygens**			
O4	-0.32835	-0.33762	-0.41193
O13	-0.32835	-0.39764	-0.41206
O16	-0.38528	-0.39102	-0.40923
O19	-0.30543	-0.33206	-0.40783
O22	-0.30543	-0.39667	-0.40750
O25	-0.38528	-0.40118	-0.40908
Terminal $O_{average}$	-0.33969	-0.37603	-0.40961
**TRIOL Ligand-Bonded Oxygens**			
O5	-0.58217	-0.58319	-0.62518
O7	-0.61519	-0.63623	-0.63616
O9	-0.58217	-0.62175	-0.62350
O10	-0.61519	-0.62413	-0.63620
O17	-0.57564	-0.61763	-0.63207
O20	-0.57564	-0.59105	-0.63336
Ligand $O_{average}$	-0.59100	-0.61233	-0.63108

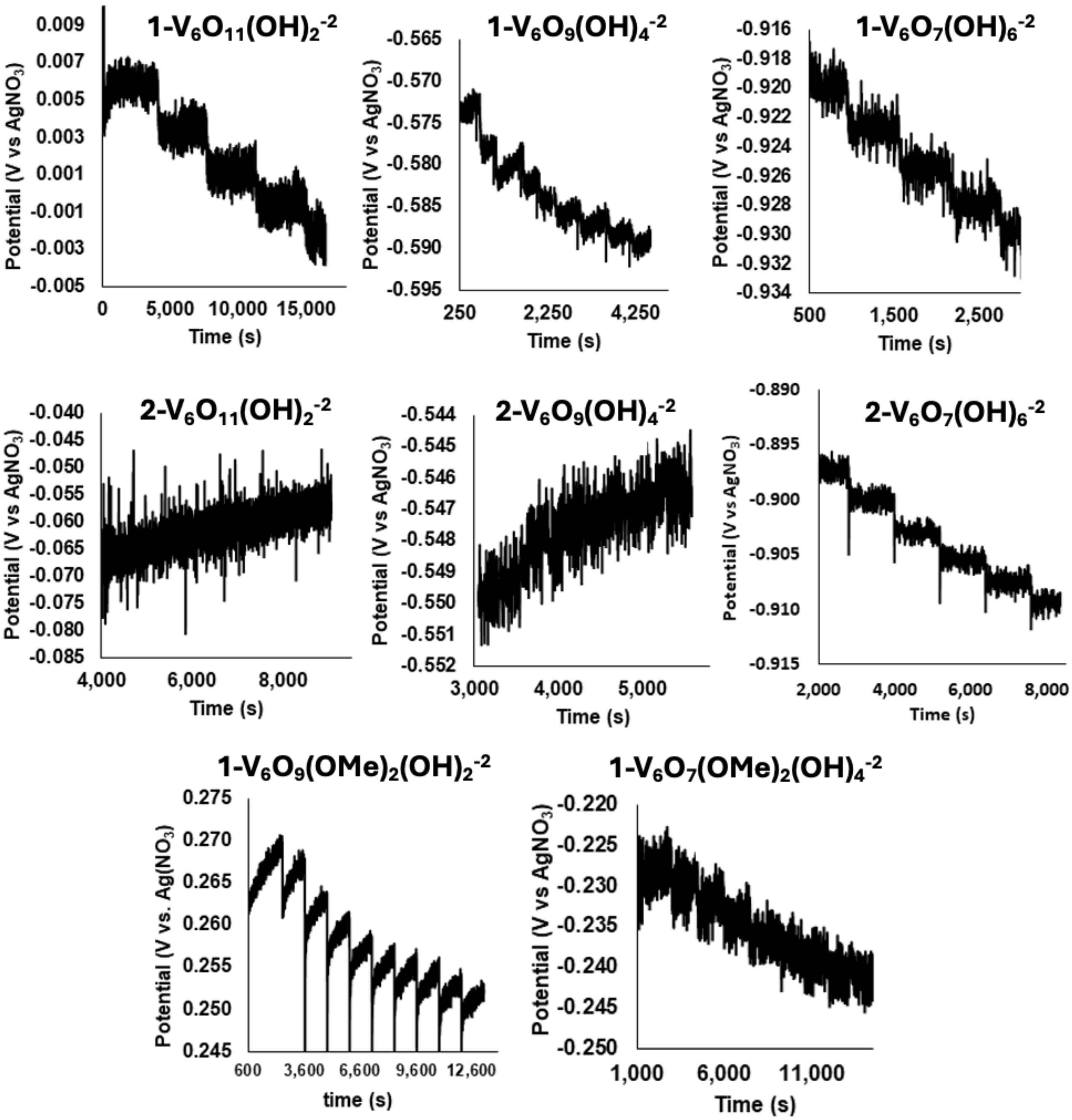

Figure S30. Open circuit potential was measured for each pair of clusters differing by two protons (i.e. 1-$V_6O_{11}(OH)_2$ and 1-V_6O_{13}) using a three electrode set-up with carbon working electrode, platinum counter electrode and a Ag/Ag(NO_3) reference electrode. At time zero the CV cell contained 0.05M buffer and 0.1M supporting electrolyte ([$^nBu_4N$][PF_6]), 0.25mM cluster 1 and 0.50mM cluster 2. Open circuit potential was tracked vs time and as the system equilibrated (slope <0.005mV/s) 100ul of cluster 1 was injected into the CV cell by an automated syringe pump (NE-1000). These injections were continued for 5-12 repetitions for each set of clusters.

References

1. Yu, H.-Z.; Yang, Y.-M.; Zhang, L.; Dang, Z.-M.; Hu, G.-H., Quantum-Chemical Predictions of pKa's of Thiols in DMSO. *J. Phys. Chem. C* **2014,** *118*, 606-622.
2. Maran, F.; Celadon, D.; Severin, M. G.; Vianello, E., Electrochemical determination of the pKa of weak acids in N,N-dimethylformamide. *J. Am. Chem. Soc.* **1991,** *113*, 9320-9329.
3. Sofja Tshepelevitsh, A. K., Märt Lõkov, Ivari Kaljurand, Jaan Saame, Agnes Heering, Paul G. Plieger, Robert Vianello, Ivo Leito, On the Basicity of Organic Bases in Different Media. *Eur. J. Org. Chem.* **2019,** 6735-6748.
4. Huffman, L. M.; Casitas, A.; Font, M.; Canta, M.; Costas, M.; Ribas, X.; Stahl, S. S., Observation and mechanistic study of facile C-O bond formation between a well-defined aryl-copper(III) complex and oxygen nucleophiles. *Chem.* **2011,** *17*, 10643-10650.
5. Agnes Kütt, S. T., Jaan Saame, Märt Lõkov, Ivari Kaljurand, Sigrid Selberg, Ivo Leito Strengths of Acids in Acetonitrile. *Eur. J. Org. Chem.* **2021,** 1407-1419.
6. Bordwell, F. G.; Branca, J. C.; Bares, J. E.; Filler, R., Enhancement of the equilibrium acidities of carbon acids by polyfluoroaryl substituents. *J. Org. Chem.* **1988,** *53*, 780-782.
7. Fertig, A. A.; Brennessel, W. W.; McKone, J. R.; Matson, E. M., Concerted Multiproton-Multielectron Transfer for the Reduction of O_2 to H_2O with a Polyoxovanadate Cluster. *J Am. Chem. Soc.* **2021,** *143*, 15756-15768.
8. Fertig, A. A.; Matson, E. M., Connecting Thermodynamics and Kinetics of Proton Coupled Electron Transfer at Polyoxovanadate Surfaces Using the Marcus Cross Relation. *Inorg. Chem.* **2023,** *62*, 1958-1967.
9. Hickey, D. p.; Minteer, S.D. Coupling Theory to Electrode Design for Electrocatalysis. *ACS Central Science* **2019**, *5*, 745-746.
